# The tapetal tissue is essential for the maintenance of redox homeostasis during microgametogenesis in tomato

**DOI:** 10.1111/tpj.16014

**Published:** 2022-11-19

**Authors:** Blanca Salazar‐Sarasua, María Jesús López‐Martín, Edelín Roque, Rim Hamza, Luis Antonio Cañas, José Pío Beltrán, Concepción Gómez‐Mena

**Affiliations:** ^1^ Instituto de Biología Molecular y Celular de Plantas (Consejo Superior de Investigaciones Científicas‐Universitat Politècnica de Valencia) C/Ingeniero Fausto Elio s/n Edif. 8E Valencia 46022 Spain

**Keywords:** *Solanum lycopersicum*, tapetum, pollen, ROS, *TPD1*, anther, male sterility

## Abstract

The tapetum is a specialized layer of cells within the anther, adjacent to the sporogenous tissue. During its short life, it provides nutrients, molecules and materials to the pollen mother cells and microsporocytes, being essential during callose degradation and pollen wall formation. The interaction between the tapetum and sporogenous cells in *Solanum lycopersicum* (tomato) plants, despite its importance for breeding purposes, is poorly understood. To investigate this process, gene editing was used to generate loss‐of‐function mutants that showed the complete and specific absence of tapetal cells. These plants were obtained targeting the previously uncharacterized *Solyc03g097530* (*SlTPD1*) gene, essential for tapetum specification in tomato plants. In the absence of tapetum, sporogenous cells developed and callose deposition was observed. However, sporocytes failed to undergo the process of meiosis and finally degenerated, leading to male sterility. Transcriptomic analysis conducted in mutant anthers lacking tapetum revealed the downregulation of a set of genes related to redox homeostasis. Indeed, mutant anthers showed a reduction in the accumulation of reactive oxygen species (ROS) at early stages and altered activity of ROS‐scavenging enzymes. The results obtained highlight the importance of the tapetal tissue in maintaining redox homeostasis during male gametogenesis in tomato plants.

## Significance Statement

During anther development, the tapetal cells that surround the sporocytes have essential roles in male meiotic progression and the formation of mature pollen grains. In tomato, despite its importance for breeding purposes, the interaction between the tapetum and sporogenous tissues is poorly understood. Here we report the generation of tomato plants devoid of tapetum by the mutation of a previously uncharacterized gene *Solyc03g097530* (*SlTPD1*). Transcriptomic and physiological analyses of the mutant plants revealed the critical role of the tapetal tissue in maintaining redox homeostasis during male gametogenesis.

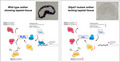

## INTRODUCTION

Sexual reproduction in both animals and plants requires the formation of haploid gametes in a complex and highly regulated process. Quite unlike animals, in flowering plants the gametes are produced post‐embryonically within specialized organs: the ovary and the anther. The female gametophyte (embryo sac) is produced from a germline originated in the ovules inside the ovary, whereas the male gametophytes (pollen) originate inside the anther. The formation of gametes in plants occurs late in development and does not depend on meristems but on cell‐to‐cell communication or tissue interactions.

The anther has a relatively simple morphological structure, and the high accessibility of the organ has resulted in the anther being the object of numerous studies on the sexual reproduction of plants. Shortly after anther primordia initiation, several somatic and germinal cells originate. Typically, the primordia contain three cell layers (L1–L3) that will develop into the external epidermis, the archesporial cells, and the inner vascular and connective tissue (Åstrand et al., 2021; Gómez et al., 2015). Archesporial cells further differentiate into three additional layers of somatic tissue: the endothecium, the middle layer and the tapetum, and a layer of microsporocytes (pollen mother cells, PMCs).

Most of the genetic information on male gametogenesis was obtained in the model plant *Arabidopsis thaliana* and two monocot crops, *Oryza sativa* (rice) and *Zea mays* (maize) (Chang et al., 2011; van der Linde & Walbot, 2019). In Arabidopsis, tapetal cell formation requires the joined action of EXCESS MICROSPOROCYTES 1/EXTRA SPOROGENOUS CELLS (EMS1/EXS), a putative Leucine‐rich repeat (LRR) receptor kinase (Canales et al., 2002; Zhao et al., 2002) and its ligand, the small peptide TAPETUM DETERMINANT 1 (TPD1) (Huang, Zhang, et al., 2016; Yang et al., 2003). In rice, a similar receptor/ligand complex is encoded by the *MULTIPLE SPOROCYTE 1* (*MSP1*) and *TDL1A* (*TPD1‐like 1A*) genes (Nonomura et al., 2003; Zhao et al., 2008), whereas in maize a *TPD1* homolog, the *MULTIPLE ARCHESPORIAL CELLS 1* (*MAC1*)/*MIL2* gene (Hong, Tang, Shen, et al., 2012; Wang et al., 2012), was identified. Downstream of this complex, several genes such as *BRI1 EMS SUPPRESSOR* (*BES1*), *DYSFUNCTIONAL TAPETUM 1* (*DYT1*), *DEFECTIVE IN TAPETAL DEVELOPMENT AND FUNCTION1* (*TDF1*) and *MYB33/65* are required for early tapetal development and function in Arabidopsis (Chen et al., 2019; Gu et al., 2014; Millar & Gubler, 2005; Zhu et al., 2008). At late stages, *MALE STERILITY1* (*MS1*) and *AMS* are involved in pollen formation and maturation (Ito & Shinozaki, 2002; Sorensen et al., 2003). Despite small differences, extensive research in Arabidopsis and rice suggests that the genetic pathway controlling tapetum development is highly for in plants (Lei & Liu, 2020; Wilson & Zhang, 2009; Zhang & Yang, 2014).

In *Solanum lycopersicum* (tomato), male sterility is a desirable trait to be used in hybrid seed production and cross‐breeding programs. Over 50 male sterile mutants were isolated more than two decades ago (Gorman & McCormick, 1997), and yet only a limited number of genes involved in male gametogenesis have been identified. Mutations in the tomato *SPOROCYTELESS/NOOZLE* ortholog prevent the formation of both male and female sporocytes and the plants are fully sterile (Hao et al., 2017; Rojas‐Gracia et al., 2017). Downstream of this gene, the *Ms10* ^*35*^ gene (*DYT1* homolog) encodes a basic helix–loop–helix (bHLH) transcription factor specifically expressed in tapetal cells and meiocytes (Jeong et al., 2014). Another bHLH protein (Solyc01g081100) has been proposed as the best candidate to encode the tomato *Ms32* gene (Liu et al., 2019). The *Solyc01g081100* gene is a homolog of the Arabidopsis *bHLH10/89/90* gene that, together with DYT1‐MYB35, forms a regulatory module during tapetum and pollen development (Cui et al., 2016). Recently, an *ABORTED MICROSPORES* (*AMS*) tomato homolog was identified that is essential for pollen development in this species (Bao et al., 2022).

Overlapping with the genetic network orchestrating anther development, additional factors and signaling molecules participate in the communication between the somatic and sporogenous tissues. These factors include hormones, secreted proteins, microRNAs (miRNAs) and cellular redox state (Dukowic‐Schulze & van der Linde, 2021). Interestingly, although a high concentration of reactive oxygen species (ROS) causes irreversible DNA damage and cell death, at low levels ROS act as signaling molecules determining cell division and cell fate (Kelliher & Walbot, 2012; Yang et al., 2018). Several lines of evidence support the hypothesis that cellular redox state is an important morphogenetic factor controlling cell differentiation and proliferation during anther development in rice (Yu & Zhang, 2019). In tomato anthers, the modulation of ROS levels achieves the proper timing of programmed cell death (PCD) in tapetal cells (Yu et al., 2017), an essential process during pollen formation.

The tapetum layer, adjacent to the developing microsporocytes, has a central role during pollen development, and its premature or delayed degradation results in pollen abortion and male sterility (Bai et al., 2019; Liu et al., 2018). In this work, we studied the molecular and physiological changes associated with the absence of tapetum and its effect on pollen formation in tomato plants. In the absence of information concerning genes conferring tapetal cell identity, we looked for candidate genes based on gene homology. We identified the *Solyc03g097530* gene as the closest homolog of the protein peptides *TPD1*, *TDL1A* and *MAC1*, and then mutant plants were produced using CRISPR/Cas9 technology. Mutant plants devoid of tapetum were used to gain insight into the genetic network and molecular signals involved in pollen formation in tomato plants. Our results provide evidence for a role of the tapetum in the modulation of redox homeostasis during early stages of pollen development.

## RESULTS

### Identification of the tomato *Solyc03g097530* gene as a candidate gene to specify tapetum identity

Previous studies in Arabidopsis, maize and rice established that tapetum identity in these species is determined by the combined function of an LRR receptor kinase and a small protein ligand encoded by the gene homologs *TPD1*, *TDL1A* and *MAC1* (Wang et al., 2012; Yang et al., 2003; Zhao et al., 2008). Following a gene homolog strategy, we looked for a tomato gene candidate to be involved in the specification of tapetum identity. The *TPD1*, the second option sequence was used as a bait in the Plant Comparative platform Phytozome (https://phytozome‐next.jgi.doe.gov) (Goodstein et al., 2012) against the tomato genome, and two homologous sequences were identified: *Solyc03g097530* and *Solyc11g012650*. The expression of these genes was analyzed in vegetative tissues (leaves) and flower buds using quantitative real‐time polymerase chain reaction (qRT‐PCR). The results showed that *Solyc11g012650* was preferentially expressed in leaves, whereas *Solyc03g097530* was expressed in developing flowers, reaching the highest level in flowers at anthesis (Figure S1). Phylogenetic analyses were performed using a list of homolog genes from different plant species obtained in a BLAST search using the *TPD1* gene (*At4g24972*) as a bait. These sequences also included the Arabidopsis closest homolog *At1g32583* and the rice ortholog *OsTDL1A* (Zhao et al., 2008). In the phylogenetic tree, *Solyc03g097530* grouped with *TPD1* and related *TPD1‐like* homologs from *Solanaceae* (Figure S1).

**Figure 1:**
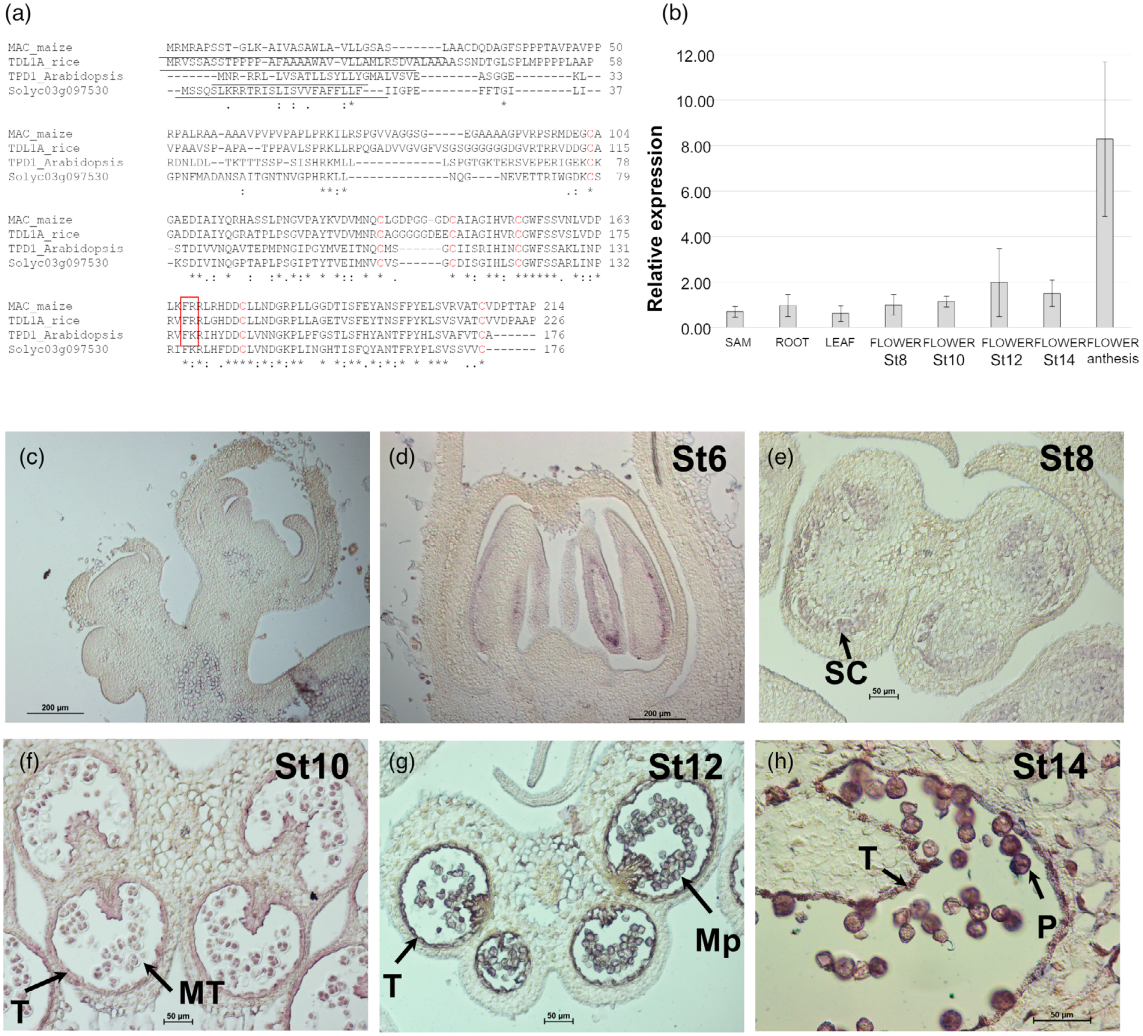
*Solyc03g097530* encodes the ortholog of *TPD1*, *TDL1A* and *MAC1* in tomato. (a) Amino acid sequence alignment between the Arabidopsis, rice, maize and tomato gene homologs. The putative signal peptides are underlined, the six conserved cysteine residues are in bold and the potential dibasic cleavage site is highlighted with a red rectangle. (b) Relative expression of *Solyc03g097530* in different plant tissues analyzed by qRT‐PCR. Data were normalized to the expression of the *SlACT10* gene and correspond to the mean (±SD) of three biological replicates. (c–h) Localization of *Solyc03g097530* transcript by *in situ* hybridization on reproductive meristems and developing flowers. Abbreviations: Mp, microspores; MT, microspore tetrads; P, mature pollen; SC, sporogenous cells; St6, floral stage 6; St8, floral stage 8; St10, floral stage 10; St12, floral stage 12; St14, floral stage 14; T, tapetum.

The protein sequence of Solyc03g097530 (176aa) was aligned with Arabidopsis TPD1 and two protein homologs that have been functionally characterized: TDL1A from rice (Zhao et al., 2008) and MAC1 from maize (Wang et al., 2012). These proteins showed high amino acid identity, mainly in the C‐terminal region, with six highly conserved cysteine residues and a putative dibasic cleavage site (Figure 1a). In addition, the Solyc03g097530 protein and its homologs contain a predicted signal peptide at their N‐terminal regions (Figure 1a, underlined). The subcellular location of the protein was determined by fusing the yellow fluorescent protein (YFP) to the C‐terminal end of Solyc03g097530, transiently expressed in *Nicotiana benthamiana* leaves. The control protein (35S:GFP) exhibited both cytoplasmic and nuclear localization (Figure S2), whereas the Solyc03g097530‐YFP protein was localized in the proximity of the plasma membrane, where it formed small dots, and in the cytosol, as large aggregates (Figure S2).

The expression of *Solyc03g097530* was analyzed in different plant tissues, including seedlings (apical and basal regions), leaves and developing flowers, using qRT‐PCR. The gene was expressed in all the tissues analyzed, reaching the highest level in flowers at anthesis (Figure 1b). The spatial and temporal expression pattern of *Solyc03g097530* was evaluated during flower development using *in situ* hybridization (Figure 1c–h). *Solyc03g097530* RNA was not detectable in inflorescence meristems and flower buds before differentiation of the anther primordium (Figure 1c). Expression was first detected at floral stage 6 at the internal layers of the developing anther that will generate the sporogenous tissue (Figure 1d,e). Later, at the tetrad stage, *Solyc03g097530* transcript was localized at the tapetum and the microspores, which were still surrounded by the callose wall (Figure 1f). The expression of the gene continues during the following floral stages in the tapetal cells that gradually disintegrated and in the pollen grains (Figure 1g,h). On the ovary, we detected transient expression in the ovule primordia of flowers at stage 8 (Figure S3).

To evaluate the conservation of Solyc03g097530 function during the development of the anther, we designed an experiment to complement a loss‐of‐function *tpd1* mutant. We used a mutant line (N843482, *tpd1* mutant) that contains a T‐DNA insertion in the *TPD1* gene. Mutant plants were indistinguishable from the wild type except that the anthers did not produce pollen grains (Figure S4). To complement the mutant phenotype, we generated a genetic construct by fusing 2.7 kb of the promoter region of Arabidopsis *TPD1* and the coding sequence of the *Solyc03g097530* gene that was used to genetically transform heterozygous *tpd1* plants. We obtained 32 independent transformants and four of them were homozygous for the mutation. These four plants produced viable pollen (Figure S4) and seeds, and hence recovered fertility, demonstrating the ability of the Solyc03g097530 protein to replace TPD1 function.

Altogether, these results suggest that *Solyc03g097530* (from now on *SlTPD1*) is the ortholog of *TPD1*, *TDL1A* and *MAC1* genes, and therefore is a good target to generate tomato plants devoid of tapetum.

### *Sltpd1* mutants specifically lack tapetal cells and are male sterile

To demonstrate the function of *SlTPD1* in the control of tapetum specification, tomato lines with mutations targeted to the third exon of the gene were generated using CRISPR/Cas9 (Figure S5). Among the T_0_ generation, we selected six diploid plants that showed percentages of edition over 80% and that mostly contained biallelic mutations (Figure S5). All the plants showed complete male sterility and developed seedless (parthenocarpic) fruits. Histological sections of the mature anthers revealed collapsed locules containing dense debris, but they did not contain viable pollen (Figure S5). In these plants, we observed a strong correlation between male sterility and the development of parthenocarpic fruits (Figure S5). F_2_ plants were obtained after pollination with wild‐type pollen, and stable single‐mutation lines were obtained. Two mutant lines (*Sltpd1* ^*Del5*^ and *Sltpd1* ^*Del2*^) containing deletions of five and two nucleotides, respectively, were chosen for further analysis. During the design of the gRNA guide, the uncharacterized *Solyc09g089880* gene was identified as a potential off‐target, although the designed guide showed four mismatches. To discard the edition of this gene in the selected lines, we amplified by PCR a fragment of the *Solyc9g089880* gene using genomic DNA from these plants (*Sltpd1* ^*del2*^ and *Sltpd1* ^*del5*^ mutants), flanking the putative target site. We found no changes in the sequence of the putative off‐target gene.

**Figure 2:**
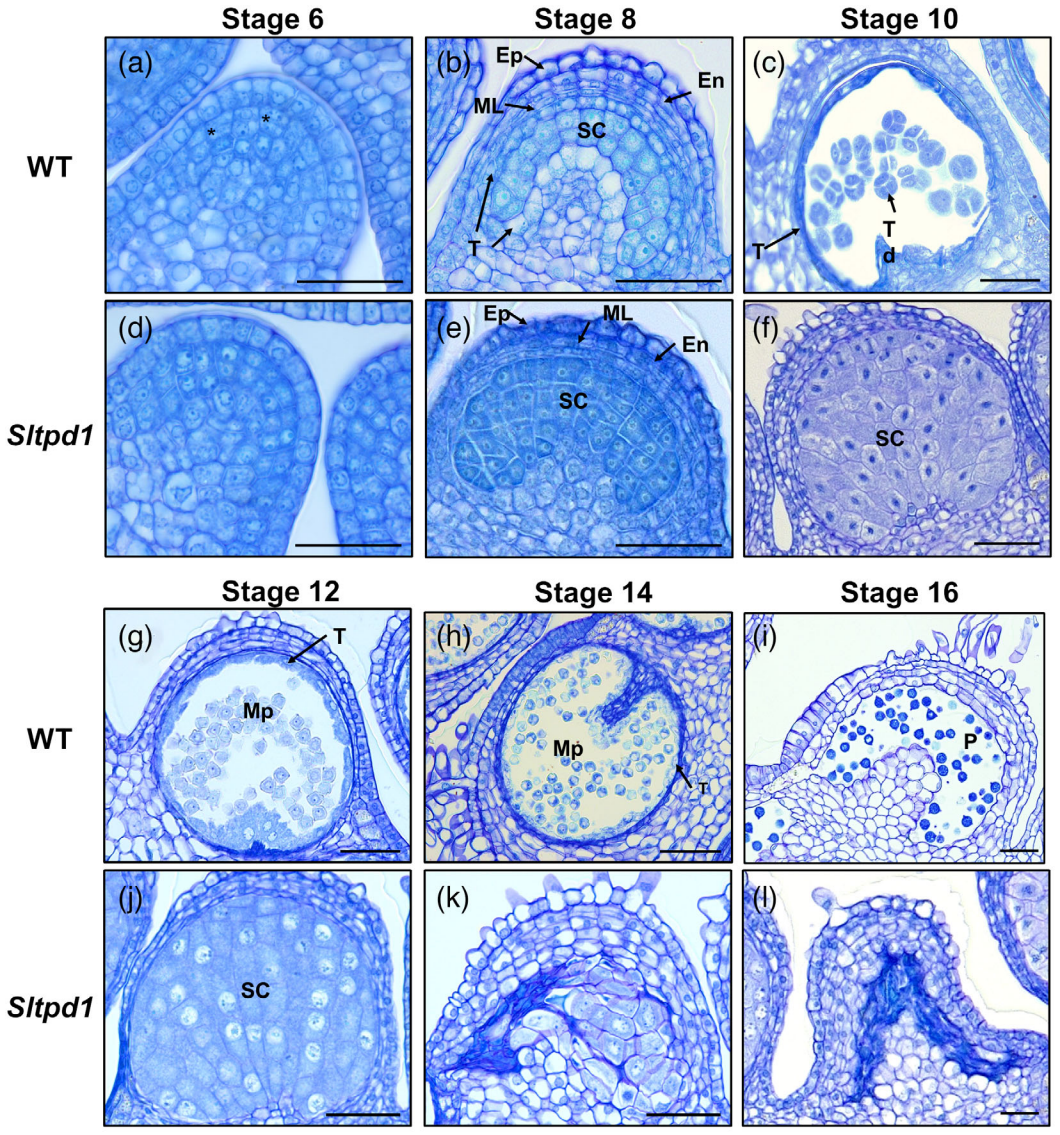
Comparison of wild‐type and *Sltpd1* mutant anther and pollen development. Histological sections of anthers from the wild type (a–c and g–i) and *Sltpd1* (*Sltpd1* ^*Del5*^) mutant (d–f and j–l) at different developmental stages. Cross section of anthers from floral stage 6 (a) and (d), floral stage 8 (b) and (e), floral stage 10 (c) and (f), floral stage 12 (g) and (j), floral stage 14 (h) and (k) and floral stage 16 (i) and (l). Floral stages have been named according to Brukhin et al., 2003. Scale bar: 50 μm. Abbreviations: Ep, epidermis; En, endothecium; ML, middle layers; Mp, microspores; P, mature pollen; SC, sporogenous cells; T, tapetum; Td, tetrads. The asterisks (*) in panel (a) mark periclinal cell divisions.

To establish the role of the *SlTPD1* gene during male gametogenesis, we compared the development of anthers from the wild type and the *Sltpd1* mutants. In wild‐type tomato anthers, cells from the L2 layer differentiate into archesporial cells that undergo periclinal divisions (parallel with the epidermis) (Figure 2a). In the *Sltpd1* mutant, anther development was slightly different to that in the wild type, showing cells with squared rather than rectangular shape and a reduced number of periclinal divisions (Figure 2d). From stage 8, we observed clear differences between the two genotypes. Whereas epidermis, endothecium and middle cell layers were formed, tapetum was not present in the mutant, and sporogenous cells seemed more abundant and disorganized compared with the wild type (Figure 2e). At stage 10, wild‐type microsporocytes completed meiosis and formed tetrads surrounded by callose and separated from the adjacent cell layers (Figure 2c). Eventually, callose was degraded, releasing the microspores that continued to develop into mature pollen grains during floral stages 12–16 (Figure 2g–i). Simultaneously, the tapetum started to degrade and was not visible by stage 16 (Figure 2i). In the mutant anthers, microsporogenous cells continued to divide and enlarge in size (Figure 2f). After extra rounds of divisions, the cells occupied the complete cavity of the locule (Figure 2j). Cell counting showed that by floral stage 8, the number of sporogenous cells in *Sltpd1* anther locules was roughly double that of the wild‐type anthers (24.0 ± 5.8 versus 49.0 ± 4.7 cells per locule section; *P* < 0.001, Student's *t*‐test). At stage 10, sporogenous cell number further increased (66.1 ± 11.3 cells per locule section; *P* < 0.001, Student's *t*‐test), and cells seemed to have initiated meiosis but failed to complete it (Figure 2j). Finally, cells degenerated causing the collapse of the anther locules and the deposition of dense cell debris (Figure 2l).

**Figure 3:**
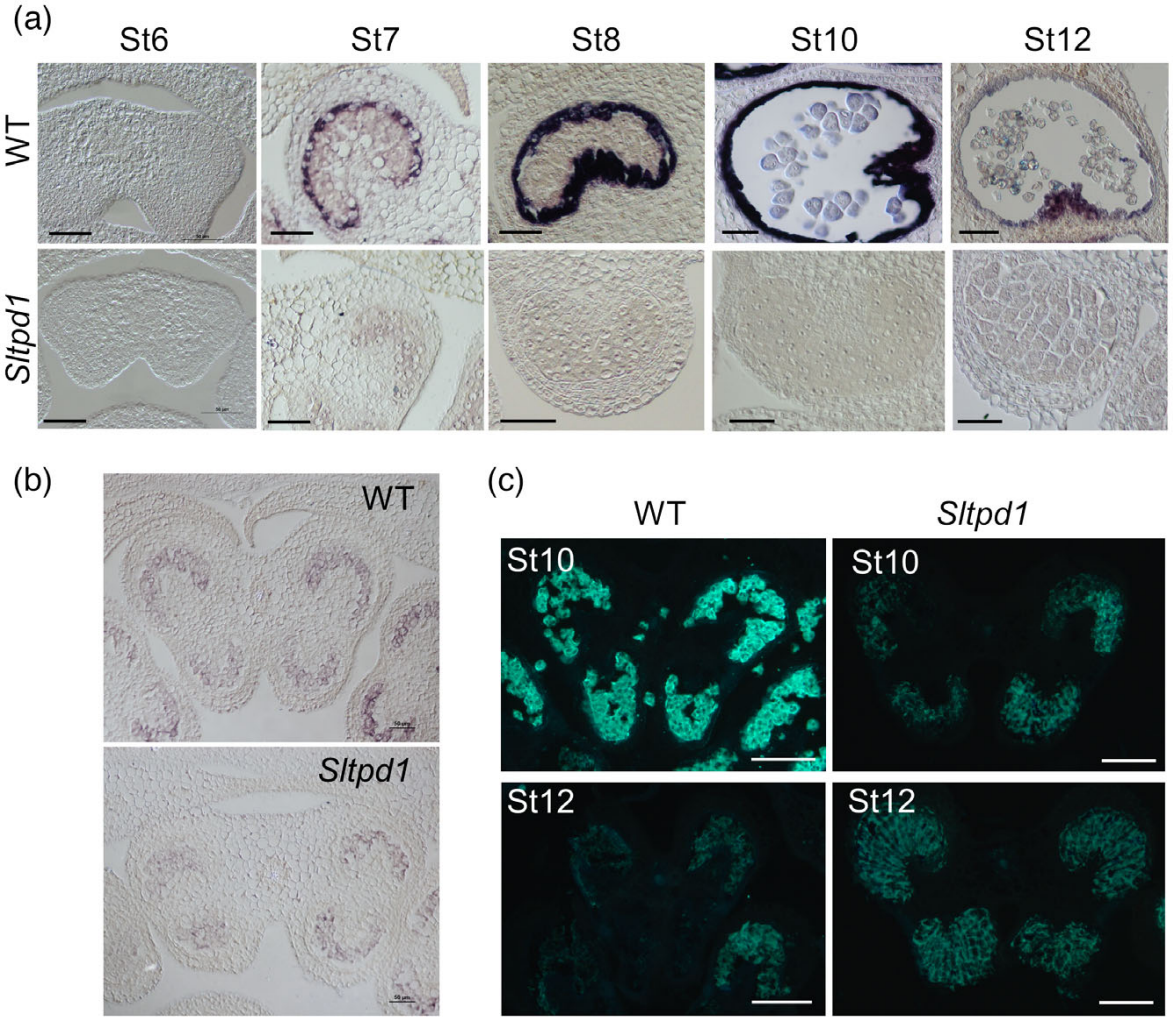
*Sltpd1* mutant anthers specifically lack tapetal cells. (a) *In situ* hybridization of the tapetum marker *TomA5B* in wild‐type and *Sltpd1* (*Sltpd1* ^*Del5*^) anthers. (b) *In situ* hybridization of the meiosis marker *SlSDS* in wild‐type and *Sltpd1* (*Sltpd1* ^*Del5*^) anthers at floral stage 8. (c) Callose deposition in anthers as observed by aniline blue staining of the wild type and *Sltpd1* (*Sltpd1* ^*Del5*^) at floral stages 10 and 12. Abbreviations: St6, floral stage 6; St7, floral stage 7; St8, floral stage 8; St10, floral stage 10; St12, floral stage 12. Scale bars: 50 μm in (a) and (b); 100 μm in (c).

The presence of tapetal tissue and meiocytes was tested by performing *in situ* hybridization assays using probes for the tapetum‐specific *TomA5B* (*Solyc01g086830*) gene (Aguirre & Smith, 1993) and the tomato homolog (*Solyc04g008070*) of the meiosis marker *SOLO DANCER* (*SDS*) gene (Azumi et al., 2002). In the wild‐type anther, the *TomA5B* probe is first visible at stage 7 and is strongly hybridized with the tapetal cells at floral stages 8 and 10 (Figure 3a). The signal decreased dramatically by stage 12, when tapetum degeneration starts (Figure 3a). In the *Sltpd1* mutant anthers no signal was obtained in any of the floral stages analyzed (Figure 3a), confirming the complete absence of tapetum in the mutant plants. In the case of the *SlSDS* probe, the hybridization signal was first observed in the wild type at floral stage 8, overlapping with the initiation of meiosis (Figure 3b). A similar result was obtained in the mutant anther, detecting the expression of the gene at floral stage 8 (Figure 3b). These results indicate that meiosis was initiated in the mutant anthers, although it failed to progress to the tetrad stage.

Callose deposition occurs around the sporogenous cells prior to meiosis initiation and later, between meiotic products (Jaffri & MacAlister, 2021). After the completion of meiosis, callose is quickly degraded after the release of callases (β‐1,3‐glucanases) by the tapetum. Using aniline blue staining, the pattern of callose deposition and degradation was analyzed in the mutant plants. In wild‐type anthers, the deposition of callose appears as an intense fluorescence signal around the tetrads that quickly disappears at the termination of meiosis (Figure 3c). In the mutant plants, the accumulation of callose was observed as a diffuse signal surrounding the sporogenous cells, and the fluorescence signal persisted in time until the collapse of the anther locule (Figure 3c).

### Identification of global transcriptional changes associated with the absence of tapetum

**Figure 4:**
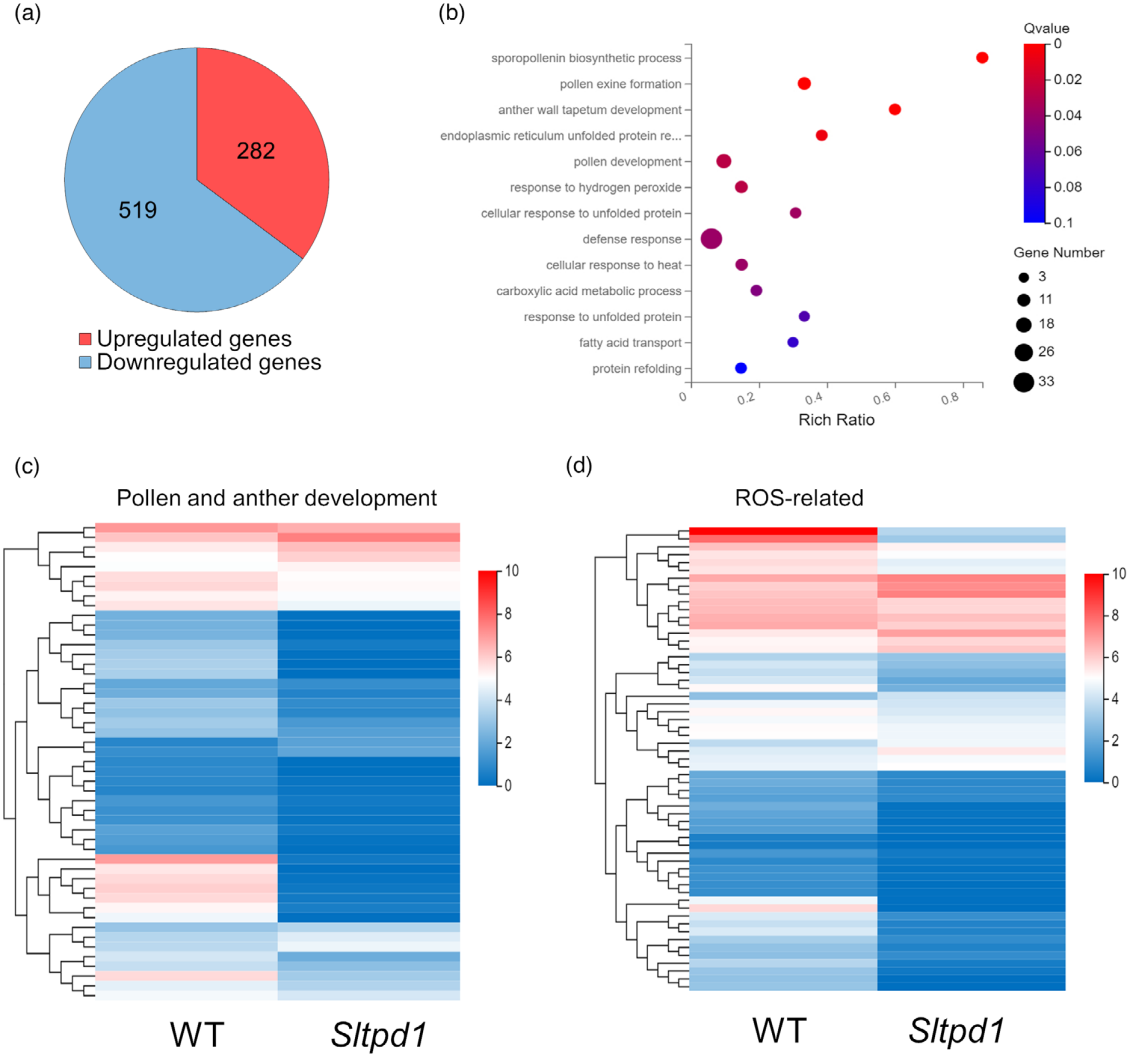
Global gene expression changes in the anthers of *Sltpd1* (*Sltpd1* ^*Del5*^) mutants at floral stage 8 in comparison with the wild type. (a) Total number of differentially expressed genes (DEGs) between wild‐type and mutant anthers. (b) GO biological process enrichment analysis. (c) Expression heat map of DEGs involved in pollen and anther development. (d) Expression heat map of differentially expressed ROS‐related genes. *Q* < 0.05; *P* < 0.05.

To identify the transcriptional changes associated with the absence of tapetum, RNAseq analyses were performed using *Sltpd1* mutant anthers from floral stage 8 (meiotic stage). Differentially expressed genes (DEGs) were selected using *Q* > 0.1 and *P* > 0.05. From the selected genes (801), 519 correspond to downregulated genes and 282 correspond to upregulated genes (Figure 4a; Table S1).

At early floral stage 8, Gene Onthology (GO) analyses revealed enrichment in genes related to pollen and tapetum development (five out 13 categories that correspond to 49 genes; Figure 4b; Table S1). Among these genes, homologs of *AMS*, *bHLH91*, *DYT1*, and *MYB35* showed strong downregulation (Table 1). Accordingly, in Arabidopsis, DYT1 is required to activate the expression of *bHLH010/bHLH089/bHLH091* genes, which in turn facilitate DYT1 nuclear localization and promote *MYB35* expression (Cui et al., 2016). In addition, we detected the strong downregulation of genes required during late stages of pollen development, including a polygalacturonase homolog of the Arabidopsis *QRT3* gene involved in microspore separation (Rhee et al., 2003) and a fatty acid‐CoA reductase (Table 1).

**Table 1:** List of genes involved in pollen development and reactive oxygen species (ROS) homeostasis that showed downregulation in the anthers of *Sltpd1* mutants compared with the wild type at floral stage 8

Gene ID	Gene homolog	log2	*Q* value
Anther wall tapetum development			
*Solyc02g079810*	Transcription factor DYT1	−6.43964989	1.26 E‐76
*Solyc08g062780*	Transcription factor aborted microspores	−7.58989677	3.02 E‐21
*Solyc03g059200*	MYB35	−8.14777345	4.14 E‐62
*Solyc01g081100*	Transcription factor bHLH91‐like	−9.19738196	7.73 E‐64
Other genes related to pollen and anther development			
*Solyc06g074320*	Transcription factor TGA9	−0.9158745	1.48 E‐19
*Solyc12g010170*	Transcription factor bHLH66	−1.24356281	0.0094393
*Solyc03g113850*	Protein JINGUBANG	−2.52418827	2.03 E‐08
*Solyc03g117800*	ECERIFERUM 3	−2.69271178	2.41 E‐39
*Solyc06g051950*	Bobber 1	−2.94998503	3.36 E‐04
*Solyc07g063180*	Dynein light chain	−2.99548166	3.63 E‐32
*Solyc03g120650*	Pollen‐specific kinase partner protein	−3.03837744	2.86 E‐04
*Solyc01g005510*	l‐Ascorbate oxidase homolog	−3.41385131	0.0328411
*Solyc07g052300*	MYB101	−4.56110971	2.39 E‐13
*Solyc04g056360*	Probable WRKY transcription factor 3	−6.45522912	6.88 E‐24
Sporopollenin biosynthesis and pollen exine formation			
*Solyc02g068400*	Polygalacturonase QRT3	−2.224485	5.13 E‐12
*Solyc01g111070*	Type‐III polyketide synthase B	−4.43900397	6.36 E‐11
*Solyc03g051960*	Fatty acyl‐CoA reductase 2	−6.20323505	1.05 E‐08
*Solyc04g008780*	Tetraketide α‐pyrone reductase 1	−8.93478906	5.77 E‐28
*Solyc02g088710*	4‐Coumarate‐CoA ligase‐like 1	−9.02558469	1.29 E‐17
*Solyc01g090600*	Type‐III polyketide synthase A	−9.20644722	6.19 E‐11
*Solyc10g009390*	Cytochrome P450 703A2	−9.84385813	1.02 E‐17
*Solyc07g015960*	Spermidine hydroxycinnamoyl transferase	−10.5269673	3.00 E‐14
*Solyc01g010900*	Cytochrome P450 704B1	−10.8055292	2.24 E‐10
*Solyc03g053130*	Strictosidine synthase‐like 13	−11.9186345	4.75 E‐11
ROS‐related genes			
*Solyc06g074320*	Transcription factor TGA9	−0.9158745	1.48 E‐19
*Solyc02g083620*	l‐Ascorbate peroxidase 5	−0.99116313	0.0195753
*Solyc08g062450*	Class‐II small heat‐shock protein Le‐HSP17.6	−1.28889207	8.78 E‐04
*Solyc07g048070*	Cytochrome b561	−1.80370303	4.43 E‐05
*Solyc02g084780*	Peroxidase 72	−2.03908491	0.0020332
*Solyc01g104860*	Peroxidase 43‐like	−2.50327644	0.0209080
*Solyc01g090710*	cMDH (cytosolic malate dehydrogenase)	−3.30689847	2.74 E‐15
*Solyc01g005510*	l‐Ascorbate oxidase homolog	−3.41385131	0.0328411
*Solyc08g036570*	Glutaredoxin‐C9‐like (MIL1)	−3.43941376	1.62 E‐07
*Solyc03g031880*	Probable polyamine oxidase 4	−4.51662449	9.01 E‐34
*Solyc10g047110*	Peroxidase 43‐like	−4.55372011	2.86 E‐08
*Solyc08g075320*	Abscisic acid 8′‐hydroxylase 3	−4.57900097	2.79 E‐04
*Solyc03g078810*	UDP‐glycosyltransferase 76B1‐like	−4.58408135	5.30 E‐11
*Solyc07g042460*	Respiratory burst oxidase homolog protein E	−4.83360202	5.37 E‐05
*Solyc05g051730*	Monothiol glutaredoxin‐S6‐like	−4.83448598	0.0398049
*Solyc01g099620*	Respiratory burst oxidase homolog protein A	−4.90590784	2.61 E‐45
*Solyc04g080760*	Peroxidase 9	−4.98729283	4.09 E‐68
*Solyc07g052550*	Peroxidase 3‐like	−5.23971051	3.43 E‐07
*Solyc10g078670*	bZIP transcription factor TGA10‐like	−5.24607495	1.22 e‐27
*Solyc10g076190*	Peroxidase 2‐like	−6.05039275	0.0078115
*Solyc02g014730*	Cytochrome P450 86B1	−6.07748043	3.81 E‐06
*Solyc01g058520*	Peroxidase 40	−6.67248882	0
*Solyc10g009390*	Cytochrome P450 703A2	−9.84385813	1.02 E‐17

When looking at signaling pathways, an important group of redox‐related genes was observed grouped under ‘cellular response to hydrogen peroxide’ and ‘defense response’. A specific expression heat‐map analysis of redox‐related genes revealed the differential expression of 70 genes, 53 of which were downregulated and 17 of which were upregulated (Figure 4c; Table S1). Among the downregulated genes (Table 1), we detected two *Respiratory burst oxidase homolog* (*Rboh*) genes (also known as NADPH oxidases), key enzymes that catalyze the formation of ROS in plants and a glutaredoxin (GRX) that shows homology with the *MIL1* gene from rice involved in microspore development (Hong, Tang, Zhu, et al., 2012). Moreover, nine peroxidases are downregulated in the mutant anthers, including homologs of the previously characterized PRX9 and PRX40 involved in pollen development in Arabidopsis (Jacobowitz et al., 2019). Peroxidases are multifunctional proteins that catalyze the oxidation of a variety of substrates by H_2_O_2_ and act as efficient components of the antioxidative system controlling ROS.

**Figure 5:**
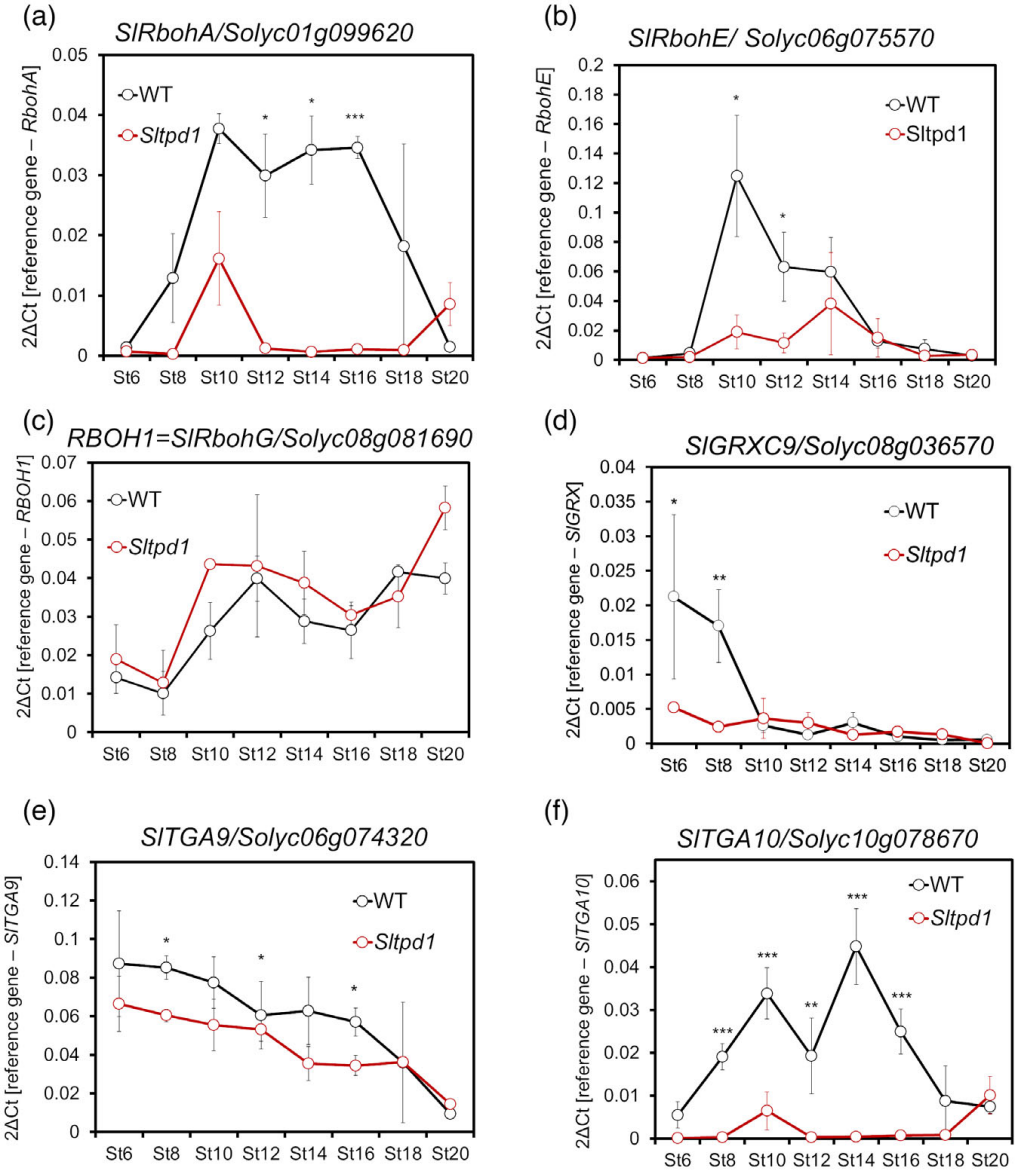
Expression pattern of genes involved in redox homeostasis during anther development in wild‐type and *Sltpd1* (*Sltpd1* ^*Del5*^) mutant plants. Quantitative RT‐PCR of (a) *SlRbohA/Solyc01g099620* gene, (b) *SlRbohE/Solyc06g075570* gene, (c) *RBOH1SlRbohG/Solyc08g081690* gene, (d) *SlGRX9/Solyc08g036570* gene, (e) *SlTGA9/Solyc06g074320* gene and (f) *SlTGA10/Solyc10g078670* gene. Data correspond to three biological replicates ± SDs. Statistical differences were inferred using a Mann–Whitney test: *P < 0.05; **P < 0.01; ***P < 0.001.

We analyzed the contribution of the genes involved in redox homeostasis to the development of the tomato anther. From the list of DEGs, a subset of key ROS‐related genes was selected, and expression levels were checked in anthers from different developmental stages (St6–St20). The expression of two tomato *RBOH* genes (*SlRbohA/Solyc01g099620* and *SlRbohE/Solyc06g075570*) was analyzed by qRT‐PCR. Moreover, we analyzed the expression of *SlRBOH1*/*SlRbohG*, recently identified as a brassinosteroid (BR)‐regulated gene involved in tapetal cell degeneration and pollen development (Yan et al., 2020). In *Sltpd1* mutant anthers, we detected an important reduction in the expression level of *SlRbohA* and *SlRbohE* at early stages of anther development (Figure 5a, b). The expression levels of *SlRBOH1*/*SlRbohG* did not significantly change during the floral stages analyzed (Figure 5c).

**Figure 6:**
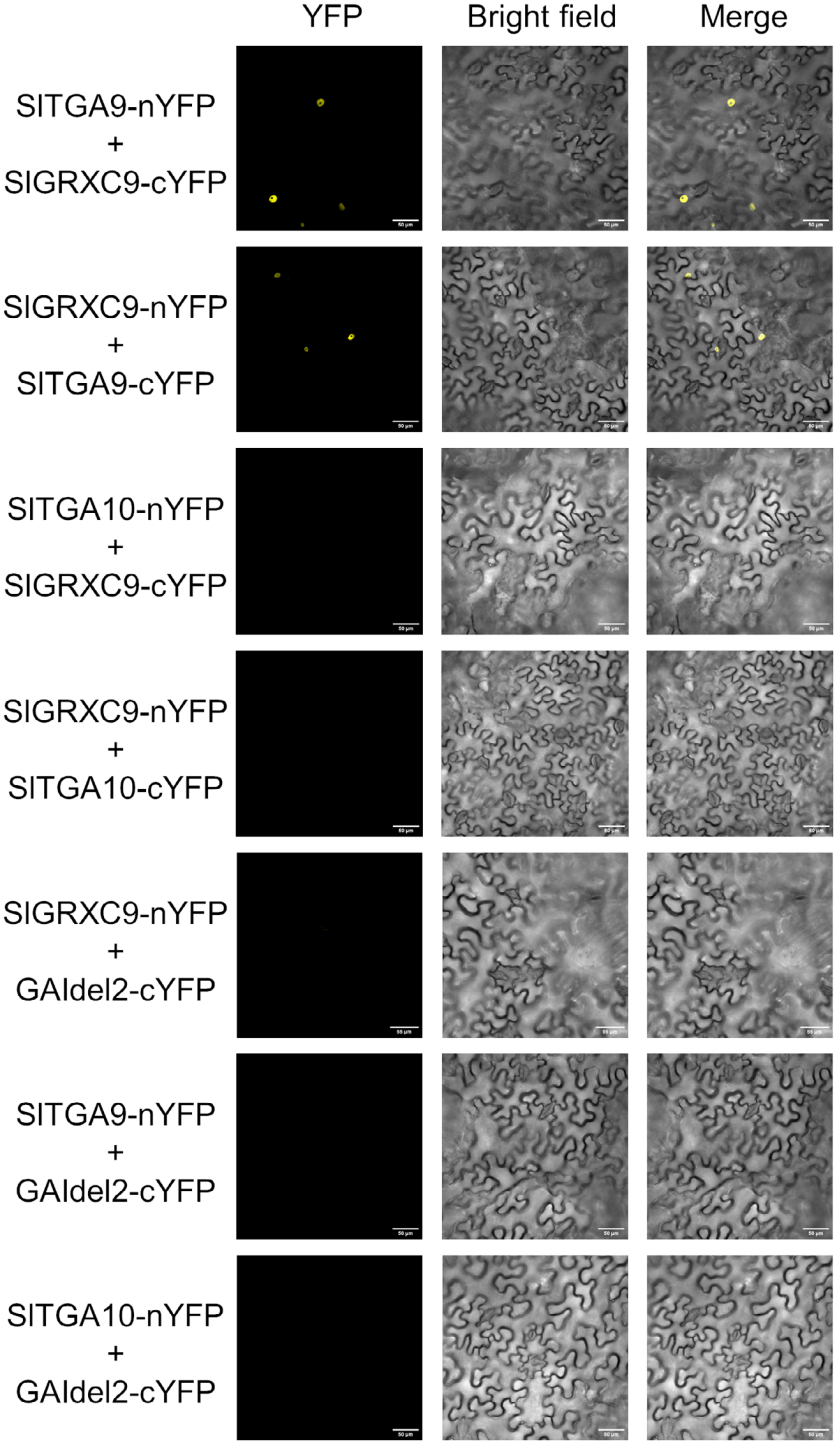
SlGRX9 physically interacts with SlTGA9, but not with SlTGA10. BiFC analysis in *Nicotiana benthamiana* leaves of SlGRX9, SlTGA9 and SlTGA10 fusions to N‐ and C‐terminal fragments of YFP observed by confocal microscopy. SlGRXC9 physically interacted with SlTGA9 in the nuclei. Representative images are shown. Del2GAI_cYFP, which does not interact with SlTGA9, SlTGA10 or SlGRXC9, was used as a negative control. Scale bars: 50 μm.

The expression of the GRX‐C9‐like gene *SlGRXC9/Solyc08g036570* was analyzed, and high levels of expression were detected in the wild type at the earliest stages analyzed (floral stages 6 and 8), whereas in the mutant samples, the expression level was greatly reduced (Figure 5a). The expression of two TGA‐like transcription factors (*Solyc06g074320/SlTGA9* and *Solyc10g078670/SlTGA10*), downregulated in the RNA‐seq, were also analyzed. Quantitative RT‐PCR experiments indicated that although *SlTGA9* showed reduced expression in the mutant at floral stages 8, 14 and 15, *SlTGA10* expression was strongly reduced in the mutant anthers from floral stage 8, and this low level persisted until floral stage 16 (Figure 5b, c). In Arabidopsis, ROXY1/ROXY2 glutaredoxins interact with TGA9/TGA10 transcription factors during another development (Murmu et al., 2010). Thus, the interaction between SlGRXC9 and SlTGA9/SlTGA10 was tested *in planta* through bimolecular fluorescence complementation (BiFC). SlGRXC9 and SlTGA9 interacted in nuclei of *N. benthamiana* leaves, but no interaction could be observed between SlGRXC9 and SlTGA10 (Figure 6).

Globally, the expression analyses suggested that the absence of tapetum is associated with the disruption of a genetic network involved in the modulation of ROS levels, especially during early stages of anther development.

### Analyses of ROS accumulation in the anther of the male sterile *Sltpd1* mutant

**Figure 7:**
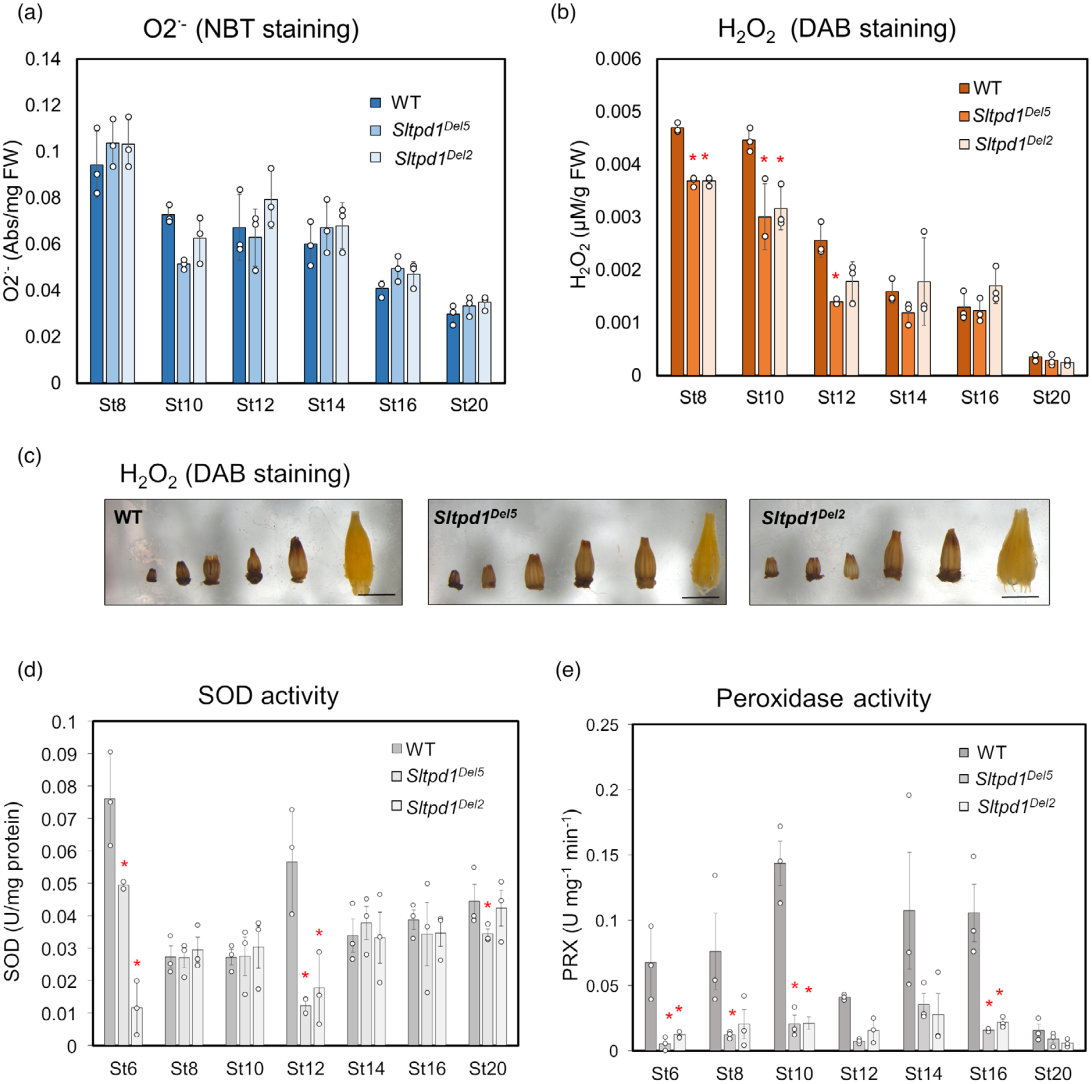
Redox homeostasis is altered in *Sltpd1* mutant anthers. Quantification of superoxide anion (O_2_·^−^) levels (a) and H_2_O_2_ levels (b) in wild‐type and *Sltpd1* anthers at different developmental stages (St8–St20). (c) DAB staining of wild‐type and *Sltpd1* anthers at the same floral stages analyzed in (a) and (b). Quantification of superoxide dismutase (d) and peroxidase (e) activity in wild‐type and *Sltpd1* flowers at different developmental stages (St6–St20). Data correspond to three biological replicates ± SDs. Statistical differences were inferred using a Mann–Whitney test: *P < 0.05; **P < 0.01.

The presence of ROS was tested in the anthers of wild‐type and *Sltpd1* mutant plants. We analyzed and quantified the presence of the superoxide anion (O_2_^.‐^) and H_2_O_2_, considered the major ROS forms in plant cells (Huang et al., 2019), using 3,3′‐diaminobenzidine (DAB) and nitroblue tetrazolium (NBT) staining, respectively. The quantification of NBT staining of the anthers, a proxy for superoxide anion presence, detected the highest levels at floral stages 8 and 10, but no differences were observed between wild‐type and mutant anthers (Figure 7a). DAB staining assays showed that in both wild‐type and mutant anthers the level of H_2_O_2_ is higher at floral stage 8 and then decreases progressively. Interestingly, at early stages (St8 and St10), the level of H_2_O_2_ was significantly lower in *Sltpd1* than in the wild type (Figure 7b, c). These results suggest that a critical H_2_O_2_ threshold should be reached during the early stages of anther development, concurring with the meiotic stage.

In plants, the maintenance of ROS levels also relies on the action of non‐enzymatic and enzymatic scavenging mechanisms. This last mechanism includes enzymes such as catalase (CAT), peroxidases (PRXs) and superoxide dismutase (SOD) (Huang et al., 2019). To study the functionality of this enzymatic scavenging mechanism in the flowers of the mutant plants, we measured SOD and PRX activities. Compared with the wild type, SOD activity showed a significant reduction in the mutant plants at floral stages 6 (pre‐meiotic), 12 and 20 (anthesis) (Figure 7d). Remarkably, PRX activity was much reduced in *Sltpd1* mutant anthers at many of the floral stages analyzed (Figure 7e). The downregulation of PRX activity at floral stage 8 was in agreement with the global downregulation of PRXs shown in the RNA‐seq experiment (Table 1).

## DISCUSSION

### *SlTPD1* is required to specify tapetum identity in tomato

The stamens are the male reproductive organs of angiosperms and the place where the pollen is produced within the flower. The different tissues that compose anthers sequentially develop from the anther primordia, suggesting that cell‐to‐cell communication is critical to coordinate growth and development (van der Linde & Walbot, 2019). The tapetum is the limiting tissue between the somatic and germinal cells, and it is in a dynamic state during its short life period, facilitating the passage of nutrients and molecules to the sporogenous cells and microspores (Pacini & Cresti, 1978).

In this study, we evince the pivotal role of the tapetal tissue during pollen development throughout the isolation and characterization of the *SlTPD1* gene. SlTPD1 shows homology with the TPD1 protein from Arabidopsis and, when transformed into the *tpd1* mutant, it was sufficient to complement the fertility defects. In addition, protein sequence alignments also showed strong domain conservation with the monocot proteins TDL1A/MIL2 and MAC1 from rice and maize. Therefore, SlTPD1 is the ortholog of the *TPD1*, *MAC1* and *TDL1A*/*MIL2* genes. On the other hand, although the putative receptor for the SlTPD1 protein has not yet been identified in tomato, our data also suggest the conservation of the receptor/ligand module in tomato plants.

In Arabidopsis, mutant plants in either *EMS1* (TPD1 receptor) or *TPD1* genes share a phenotype, the lack of tapetum and the production of extra sporocytes (Yang et al., 2003; Zhao et al., 2002). We detected the expression of *SlTPD1* by *in situ* hybridization on the anther wall early in development until late stages, where it appeared associated with the tapetum and microsporocytes. In this aspect *SlTPD1* slightly differs from *TPD1* that is preferentially expressed in microsporocytes, whereas *EMS* is predominantly expressed in the tapetum (Yang et al., 2003; Zhao et al., 2002). In maize, MAC1 is expressed early in anther ontogeny, where it suppresses archesporial cell proliferation, suggesting that cell position rather than lineage determines cell fate during anther development (Wang et al., 2012). This hypothesis is in agreement with the phenotype of *Sltpd1* mutants that showed defects in the shape and pattern of division of the archesporial cells. It has been shown that the ectopic expression of *TPD1* activates cell division, possibly by altering the expression of cell‐cycle genes (Huang, Wijeratne, et al., 2016). Taken together, we propose a dual role for *SlTPD1* in the control of archesporial cell divisions and the determination of tapetal cell identity in tomato plants.

Most *TPD1* homologs are expressed in different tissues outside the anther, including leaves, roots, seedlings (Hong, Tang, Shen, et al., 2012; Wang et al., 2012; Yang et al., 2003) and ovules (Wang et al., 2012; Yang et al., 2005). At present, a possible role of these proteins during vegetative development remains elusive. However, in monocots, *TPD1* orthologs have been reported to control megaspore mother cell proliferation during ovule development (Sheridan et al., 1996; Zhao et al., 2008). Using *in situ* hybridization, the expression of *SlTPD1* was detected in anthers and the developing ovules. *Sltpd1* mutant plants did not show obvious defects in ovule development, and flowers formed normal seeded fruits when pollinated with wild‐type pollen. A peculiar and distinctive phenotype of the tomato *Sltpd1* mutants is the formation of seedless fruits (parthenocarpic). Parthenocarpy, the formation of fruits in the absence of pollination and fertilization, is often the consequence of the precocious activation of molecular events normally triggered by these processes (Molesini et al., 2020). Also, it could be achieved by external applications of different hormones or growth regulators (Vivian‐Smith & Koltunow, 1999). In tomato plants, several reports suggest a role for developing stamens or male gametophytes in the repression of ovary growth (Hao et al., 2017; Medina et al., 2013; Okabe et al., 2019; Rojas‐Gracia et al., 2017). Mutations in *SlTPD1* caused complete male sterility and the production of small parthenocarpic fruits. This phenotype could support this repressive effect exerted by male gametogenesis progression. Alternatively, the abnormal progression of male gametogenesis could result in the production of signaling molecules that indirectly activate premature ovary growth. In this regard, antisense plants targeting *SlRBOHB*/*SlWfi1*, a tomato gene involved in the generation of ROS, show several developmental defects including parthenocarpic fruit development (Sagi et al., 2004). However, *SlRBOHB/SlWfi1* was not differentially expressed in the mutant anthers of *Sltpd1* mutants, suggesting that the specific alteration of *SlRBOH* genes could lead to similar phenotypes during reproductive development.

### Tapetal tissue is essential to maintain redox homeostasis during pollen formation

The results presented in this study show that the absence of tapetal tissue in *Sltpd1* mutants has a huge impact on the transcription of genes involved in redox homeostasis in the anther at early stages. Moreover, a reduction in ROS levels seems to be associated with the failure of pollen mother cells to progress into meiosis. In agreement with this observation, pioneering work in maize showed that hypoxia triggers meiotic fate acquisition, acting as a positional cue for germ cell production (Kelliher & Walbot, 2012).

**Figure 8:**
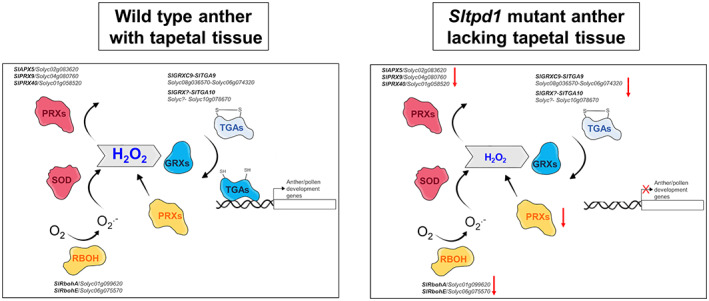
Working model summarizing the genetic elements of the redox network affected by the absence of *SlTPD1* and the concomitant tapetum loss, at early stages of tomato anther development. Enzymatic ROS accumulation (orange) is attenuated by an ROS‐scavenging mechanism (pink). Changes in ROS levels activate signaling pathways (blue) that result in the induction of genes involved in anther/pollen development. SlGRX9 physically interacts with SlTGA9 but not with SlTGA10 *in planta*. Abbreviations: APX, ascorbate peroxidase; GRXs, glutaredoxins; PRXs, peroxidases; RBOH, respiratory burst oxidase homolog; SOD, superoxide dismutase; TGAs, TGA transcription factors.

Besides the production of ROS as a product of several metabolic processes, specific enzymatic machinery is in charge of maintaining redox homeostasis in plants. ROS production in extracellular spaces partly relies on *RBOH* genes, also known as NADPH oxidases, which catalyze the generation of superoxide radicals. Enzymatic scavenging mechanisms involve CAT, PRXs and SOD, although PRXs can act as both ROS‐generating and ROS‐processing components (Mittler, 2017). Cellular changes of ROS levels can act as a signal to drive differentiation and morphogenesis during reproductive development. In Arabidopsis, *RBOHE* is specifically expressed in the tapetum, and the genetic interference with the temporal ROS pattern resulted in altered tapetal PCD and male sterility (Xie et al., 2014). In addition, PRX9 and PRX40 are extensin PRXs specifically expressed in the tapetum that act as scavenging molecules contributing to tapetal cell wall integrity (Jacobowitz et al., 2019). ROS signaling includes GRXs that act as sensors of redox status, altering signal transduction pathways that result in biological responses (Song et al., 2002). Studies in Arabidopsis, maize and rice have highlighted the importance of GRXs in the formation of the anther and the differentiation of microsporocytes (Hong, Tang, Zhu, et al., 2012; Kelliher & Walbot, 2012; Xing & Zachgo, 2008). In rice, a mutation in the anther‐specific GRX *MICROSPORELESS 1* (*MIL1*) prevents the completion of meiosis during male gametogenesis. *MIL1* encodes a CC‐type GRX that specifically interacts with TGA transcription factors (Hong, Tang, Zhu, et al., 2012). The rice glutaredoxin MALE STERILE CONVERTED ANTHER 1 (MSCA1) is required for the creation of a hypoxic niche critical for the specification of anther germinal cells (Kelliher & Walbot, 2012). In Arabidopsis, *ROXY1* and *ROXY2* also encode CC‐type GRXs and are required for the formation of the adaxial anther lobe, possibly with other GRXs or redox regulators. ROXY1 and ROXY2 proteins can interact with the TGA transcription factors TGA9 and TGA10 in *Nicotiana tabacum* (tobacco) leaves (Xing & Zachgo, 2008). These authors suggest that this interaction results in the modification of TGA9/10 and its activation as a transcriptional factor (Murmu et al., 2010). A similar genetic network to the ones described in rice and Arabidopsis should operate in tomato anthers, where ROS produced in the tapetal cells orchestrate anther wall development and the progression of pollen mother cells into meiosis. Using a tomato mutant lacking the tapetum, we identified several elements of this network that were included in the proposed working model (Figure 8). ROS produced by RBOHs (*SlRbohB* and *SlRbohE*) and PRXs results in the accumulation of H_2_O_2_ in tapetal tissue. GRXs, including *SlGRXC9*, could target TGA transcription factors (*SlTGA9* and *SlTGA10*) for activation, thus regulating the expression of a set of genes required for further stages of pollen and anther development. This genetic network is severely affected by the absence of *SlTPD1* and the concomitant loss of the tapetum, especially during early stages of anther formation. The identification of this network of genes will facilitate dissecting this regulatory mechanism controlling anther and pollen development in tomato plants.

## EXPERIMENTAL PROCEDURES

### Plant material and growth conditions

Tomato (*Solanum lycopersicum* L.) seeds from cultivar Moneymaker were grown in a glasshouse in pots with a 2:1:1 mixture of peat:vermiculite:perlite with a temperature regime of 25–30°C day and 18–20°C night. Natural light was supplemented with lamps to obtain a 16‐h light/8‐h night photoperiod. Flower samples were collected at different developmental stages according to bud size (Brukhin et al., 2003). In this study, eight floral stages were analyzed that correspond to the following sizes: St6, 0.3 cm; St8, 0.4 cm, meiotic; St10, 0.5 cm, tetrad of microspores; St12, 0.6 cm; St14, 0.7 cm; St16, 0.8 cm, pollen mitosis; St18, 0.9 cm; and St20, 1 cm, anthesis. For the characterization of tomato fruits, the size and weight of at least 30 fruits from the different genotypes were analyzed when fully ripe.

*Nicotiana benthamiana* plants were grown under the same conditions as the tomato plants. Four‐week‐old plants were used for transient expression experiments.

*Arabidopsis thaliana* Columbia (Col) plants were used as the wild‐type genotype. The *tpd1* mutant corresponds to the T‐DNA insertion line N843482 (SAIL_1174_B09) obtained from the Nottingham Arabidopsis Stock Centre (NASC, https://arabidopsis.info). The line was genotyped using the T‐DNA‐specific primer LBb1 and the gene‐specific primer pair TPD1‐LP1/TPD1‐LP2 that amplifies 427 bp from the *TPD1* gene (Table S2). Arabidopsis plants were grown in seed pots in a growth chamber with a 2:1:1 mixture of peat:vermiculite:peat at 21°C under long‐day conditions (16‐h light/8‐h dark) and irrigated with Hoagland's solution.

### Phylogenetic analysis

The phylogenetic tree was inferred by the neighbor‐joining method using Poisson‐corrected amino acid distances. A total of 1000 bootstrap pseudo‐replicates were used to estimate the reliability of internal nodes. Tree inference was performed using mega 6 (Tamura et al., 2013). The data set comprised 18 *TPD1*‐like genes from different plant species, obtained from the GenBank database (Table S1).

### Plasmid assembly

#### Construct for the complementation of the Arabidopsis *tpd1‐2* mutant

A fragment of 2.7 kb from the *TPD1* promoter (5′ region of the gene) was fused to the coding sequence of the tomato *SlTPD1* gene. First, both fragments were independently amplified by PCR and cloned into the intermediate vectors pENTRY 5′ TOPO and pCR8 (Invitrogen, now ThermoFisher Scientific, https://www.thermofisher.com), respectively. Second, a multisite gateway recombination reaction was performed to introduce both sequences into the binary destination vector pK7m24GW,3 (https://gatewayvectors.vib.be/) to obtain the final construct *pAtTPD1::SlTPD1*.

#### Design of gRNA and CRISPR/Cas9 construct for *SlTPD1* gene editing

For the design of optimal gRNAs, the target site was selected using the Breaking‐Cas design tool (Oliveros et al., 2016). Among the proposed guides, we chose the one with the highest score and with the lowest number of off‐target genes. This tool is freely available online (https://bioinfogp.cnb.csic.es/tools/breakingcas). CRISPR/Cas9 plasmid assembly was performed using the Golden Braid (GB) modular framework and tools (https://gbcloning.upv.es/). First, a single gRNA sequence was obtained by the annealing of complementary primers and then assembled with GB1001 (U626 promoter) and GB0645 (scaffold RNA) parts into the destination vector pDGB3α1. In successive multipartite GB reactions, this first module was assembled with the GB0639 and GB0226 parts (containing hCas9 and *nptII* transcriptional units, respectively) into the final destination vector. The final construct was then transformed into *Agrobacterium tumefaciens* strain LBA4404. The primers used are listed in the Table S2.

### Plant transformation

Arabidopsis transgenic plants were obtained using the floral‐dip method (Clough & Bent, 1998). Briefly, plants were grown under long‐day conditions until flower transition occurs and then the main stem was removed to allow the growing of secondary meristems. *Agrobacterium* inoculation (C58C1 strain carrying the construct of interest) was performed by immersion of the shoots (2–5 cm length) in a suspension containing 5% sucrose and 0.05% Silwet L‐77. Transformant plants were selected in the presence of kanamycin and transferred to soil for further analyses.

Tomato transformants were obtained by *in vitro* co‐cultivation of the *Agrobacterium* strain LBA4404 (carrying the binary vector of interest) and cotyledon explants (Ellul et al., 2003). Transformants were selected in the presence of kanamycin and after rooting, transferred to the glasshouse.

### Genotyping of CRISPR/Cas9 edited plants

Genomic DNA was extracted from young leaves or unopened flower buds. A 530‐bp fragment from the *SlTPD1* genomic region flanking the targeted region was amplified using oligos SlTPD1G For and SlTPD1G Rev, purified and sequenced. T_0_ plants with percentages of edition over 80% were selected using the online tool TiDE (http://shinyapps.datacurators.nl/tide/) (Brinkman et al., 2014). We then used the online software ice 2 CRISPR analysis tool (https://ice.synthego.com) to identify the number and type of edition for each plant.

For the genotyping of stable and Cas9‐free edited plants, PCR‐based molecular markers were designed. We used cleaved amplified polymorphic sequences (CAPS) markers (Konieczny & Ausubel, 1993) that detect polymorphisms that occur in restriction sites. The deletions present in the *Sltpd1* ^*del2*^ and *Sltpd1* ^*del5*^ alleles generated new restriction sites for *Bse*GI and *Nco*I enzymes, respectively. Using SlTPD1G For and SlTPD1G Rev oligos (Table S2), a 530‐bp fragment was obtained from genomic DNA. *Bse*GI generated two fragments of 308 bp and 220 bp in the *Sltpd1* ^*del2*^ allele and *Nco*I generated two fragments of 299 bp and 226 bp in the *Sltpd1* ^*del5*^ allele. Neither of the enzymes cut the wild‐type fragment.

### Subcellular localization of SlTPD1

The coding sequence of *SlTPD1* was cloned via Gateway LR reaction into the pEarleyGate101 vector (containing the YFP fluorescent tag) (Earley et al., 2006) to generate the expression vector SlTPD1‐YFP. The vector was transformed into *Agrobacterium tumefaciens* strain C58 and used to agroinfiltrate 4‐weeks‐old *N. benthamiana* leaves. After 48 h of infiltration, the localization of the fluorescence fusion protein was determined on *N. benthamiana* leaf disks by confocal scanning microscopy (LSM 780; Zeiss, https://www.zeiss.com/). A 35S:GFP construct was used as the control.

### Expression analyses by qRT‐PCR

Total RNA was extracted from frozen tissue using the E.Z.N.A. Plant RNA Kit (Omega Bio‐tek, https://www.omegabiotek.com). RNA was treated with DNAseI (ThermoFisher Scientific) to remove genomic DNA and quantified in a NanoDrop ND‐1000 Spectophotometer (ThermoFisher Scientific). For first‐strand cDNA synthesis, 1 μg of DNase‐treated RNA was used for reverse transcription using a PrimerScript RT reagent kit (TaKaRa, https://www.takarabio.com) and a mix of oligo poli‐dT and random hexamers. The resulting cDNA was used for qRT‐PCR with the MasterMix qPCR ROX PyroTaq EvaGreen 5× (CmB, https://www.cultek.com/) and the reaction was run on a QuantStudio 3 (Applied Biosystems, now ThermoFisher Scientific). Relative expression levels were calculated by normalizing to the reference genes *ACT* (Arabidopsis experiments) or *SlActin8* (tomato experiments) and using the ΔΔ^*C*t^ method. All primers showed amplification efficiencies between 90% and 110%. The primers used are listed in the Table S2.

### RNA *in situ* hybridization in tomato flowers

Fresh floral samples were fixed in FAE (4% formaldehyde, 5% acetic acid, 50% ethanol) overnight at 4°C, and afterward, stored in 70% ethanol. Samples were embedded in paraffin using an automated tissue processor (Leica TP1020; Leica Biosystems, https://www.leicabiosystems.com/).

To generate gene‐specific probes, cDNA fragments were cloned under T7/SP6 promoter sequences. For *SlTPD1* a 284‐bp DNA fragment from the 5′ coding region was amplified by PCR using cDNA from flowers and cloned into the pGEM‐T Easy vector (Promega, https://www.promega.com). For *TomA5B* and *SlSDS* genes, we used cDNA fragments of 442 and 440 bp, respectively. Digoxigenin‐labeled probes were transcribed *in vitro* with T7 or SP6 RNA polymerases. RNA was hybridized *in situ* (Gómez‐Mena & Roque, 2018; Huijser et al., 1992) in paraffin‐embedded sections (8 μm), and color was detected with 5‐bromo‐4‐chloroindol‐3‐yl phosphate/nitrateblue tetrazolium (BCIP/NBT) (Roche, https://www.roche.com).

### Histological techniques

For histological studies, tissue was fixed in FAE overnight at 4°C and stored in 70% ethanol. Samples were embedded in acrylic resin (Technovit 7100; Kulzer, https://www.kulzer.com) according to the manufacturer's instructions. For histological analysis, resin sections were stained with 0.05% toluidine blue in 0.1 m, pH 6.8, phosphate buffer (O'Brien et al., 1964) and visualized with a Leica DM 5000B microscope (Leica Microsystems) under bright field.

### Aniline blue staining in cryosections

For assays in which fresh tissue was needed, samples were fixed in NEG‐50 (Richard Alan Scientific), rapidly frozen in liquid nitrogen, and cut into 16 μm sections using a cryostat (Microm HM 520). Cryosections were stained for 10 min in the darkness with 0.5% aniline blue in 0.07 mm sodium phosphate buffer and visualized in a Leica DM 5000B microscope (Leica Microsystems, https://www.leica‐microsystems.com).

### Pollen viability assay

Alexander's staining was carried out as previously described (Peterson et al., 2010), with 2 min of incubation at 50°C on a hot plate. For pollen viability, pollen was released from the anthers by squeezing, then stained and counted. Samples were visualized in a Leica DM 5000B (Leica Microsystems) microscope under bright field. For each sample, 30 anthers from five different flowers were used.

### Histochemical localization and quantification of hydrogen peroxide (H_2_O_2_ ) and superoxide radical (O_2_ ^·‐^)

Hydrogen peroxide localization was performed in anthers obtained from flowers in different developmental stages. Immediately after dissection, anthers were submerged in a 1 mg ml^−1^ DAB‐HCl (pH 3.8) solution for 16 h under light conditions (Unger et al., 2005), then cleared in 80% ethanol for 20 min and observed in a binocular microscope (Leica Microsystems). Hydrogen peroxide levels were quantified following a similar method. After staining in DAB‐HCl and clearing with ethanol, anthers were pulverized in liquid nitrogen, dissolved in 0.2 m HClO_4_ and centrifuged at 12 000 ***g*** for 10 min. The absorbance of the supernatant was quantified at 450 nm. H_2_O_2_ concentrations were obtained through a standard curve for known hydrogen peroxide concentrations diluted with 0.2 m HClO_4_‐DAB (Kotchoni et al., 2006).

Superoxide radical was measured as formazan formation over time from tetrazolium blue. Flowers from different developmental stages were weighed, submerged in 50 mm potassium phosphate buffer (pH 7.8) containing 0.1% NBT and 10 mM sodium azide, and left to stain for 2 h before clearing in 70% ethanol. After staining, tissue was rapidly frozen in liquid nitrogen and ground. Formazan was selectively extracted using 200 μl of DMSO and absorbance was measured at 550 nm.

### Peroxidase (PRX) and superoxide dismutase (SOD) activity

Flowers at different developmental stages were collected and frozen in liquid nitrogen. Frozen tissue was ground and homogenized in extraction buffer (0.1 m Tris, pH 7.0, 0.1% ascorbic acid, 0.1% l‐cysteine, 0.5 m sucrose and 10 mg ml^−1^ PVP) and centrifuged at 4°C for 15 min, saving the supernatant. Total protein was quantified using the Bradford method (Bradford, 1976). Briefly, 10 μl of crude extract was added to a tube containing 1 ml of Bradford solution (0.01% Coomasie Brilliant Blue G‐250, 4.7% ethanol, 8.5% phosphoric acid) and mixed. After 2 min, the absorbance was measured at 595 nm. A standard curve was generated using known concentrations of BSA.

For SOD activity, 25 mg of protein from the crude extract was added to 1 ml of SOD buffer (50 mm PBS, pH 7.6, 0.01 mm EDTA, 50 mm sodium carbonate, 12 mm l‐methionine, 10 μm riboflavin, 50 μm NBT) and incubated at room temperature (22–25°C) under light conditions for 10 min. Absorbance was measured at 550 nm and SOD buffer without extract was used as a negative control. SOD activity was quantified as the quantity of enzyme required to inhibit 50% of the photoreduction of NBT.

For PRX activity, 25–75 mg of protein from the crude extract was added to 1 ml of PRX buffer (0.85 mm hydrogen peroxide in HEPES, pH 7.0, 0.125 m 4‐aminoantipyrene, 8.1 mg ml^−1^ phenol) and the change in absorbance was measured for 2 min at 510 nm. A standard curve was generated using known concentrations of horseradish peroxidase.

### RNA‐Seq analyses

Total RNA was extracted from stage‐8 stamens from frozen tissue of wild‐type and *Sltpd1* plants using a NucleoSpin RNA Plant kit (Macherey‐Nagel, https://www.mn-net.com/) and measured in a NanoDrop ND‐1000 Spectophotometer (ThermoFisher Scientific). The RNA quality was assessed based on the RNA integrity number (RIN) using a Bioanalyzer 2100 (Agilent, https://www.agilent.com/) and samples with RIN > 8 were selected for the experiment. RNA sequencing was performed using the BGISEQ Technology platform at BGI (https://www.bgi.com/global). A total of three biological replicates were used for each sample set. GO enrichment, KEGG enrichment and statistical analysis were performed through the Dr Tom platform (BGI).

### Statistical analysis

spss statistics 27 (IBM, https://www.ibm.com) was used for statistical analysis. For each data set, a Shapiro–Wilk normality test was run. For normally distributed data, a Student's *t*‐test was used for pairwise comparison. Non‐normally distributed data were analyzed with a Mann–Whitney test.

### Bimolecular fluorescence complementation (BiFC)

The coding sequences of *SlGRXC9/Solyc08g036570*, *SlTGA9/Solyc06g074320* and *SlTGA10/Solyc10g078670* were amplified by PCR and cloned into pCR8 using the pCR™8/GW/TOPO™ TA Cloning Kit (Invitrogen, now ThermoFisher Scientific). Then cDNAs were fused in frame at the N terminus with the N‐terminus half (nYFP) or C‐terminus half (cYFP) of the fluorescent YFP protein via Gateway. The vectors were transformed into *Agrobacterium tumefaciens* strain C58 and used to agroinfiltrate 4‐week‐old *N. benthamiana* leaves to a total OD_600_ of 0.1. After 72 h of infiltration, protein–protein interactions were analyzed on leaf disks by confocal scanning microscopy (LSM 780; Zeiss). Intensity was adjusted using the negative control Del2GAI_cYFP (Gallego‐Bartolomé et al., 2012), which does not interact with SlTGA9, SlTGA10 or SlGRXC9.

### ACCESSION NUMBERS

*SlTPD1* (*Solyc03g097530*); *SlSDS* (*Solyc04g008070*); *TomA5B* (*Solyc01g086830*); *SlRbohA* (*Solyc01g099620*); *SlRbohE* (*Solyc06g075570*); *RBOH1 = SlRbohG* (*Solyc08g081690*); *SlGRX9* (*Solyc08g036570*); *SlTGA9* (*Solyc06g074320*); *SlTGA10* (*Solyc10g078670*).

## AUTHOR CONTRIBUTIONS

CG‐M designed the research and wrote the grant that funded the project. BS‐S, MJL‐M, RH and ER performed the experiments. CG‐M and BS‐S wrote the article. LAC and JPB supervised the study and reviewed the article. All authors agreed with the final version for publication.

## CONFLICT OF INTEREST

The authors declare that they have no conflicts of interest associated with this work.

## Supporting information


**Figure S1.** Identification of *TPD1‐like* genes in tomato.

**Figure S2**. Subcellular localization of Solyc03g097530 (SlTPD1) protein in *Nicotiana benthamiana* leaves.

**Figure S3.** Complementation of the male‐sterile floral phenotype of the Arabidopsis *tpd1* mutant using the *Solyc03g097530* (*SlTPD1*) gene.

**Figure S4.** Expression of *Solyc03g097530* (*SlTPD1*) in the ovary detected using *in situ* hybridization.

**Figure S5.** Characterization of CRISP/Cas9‐mediated *Solyc03g097530* (*SlTPD1*) edited tomato plants.

**Table S1**. Accession numbers of *TPD1*‐like gene sequences from different plant species used for the phylogenetic analysis.
**Table S2**. Oligonucleotides used in this study.

**Table S3.** List of differentially expressed genes (DEGs) between wild‐type and *Sltpd1* mutant anthers from floral stage 8.
