# Supplementary figures and images for: The tapetal tissue is essential for the maintenance of redox homeostasis during microgametogenesis in tomato
Source: Plant J. 2022 Nov 19;112(5):1281–97. doi: 10.1111/tpj.16014 (PMC10100220; doi:10.1111/tpj.16014)

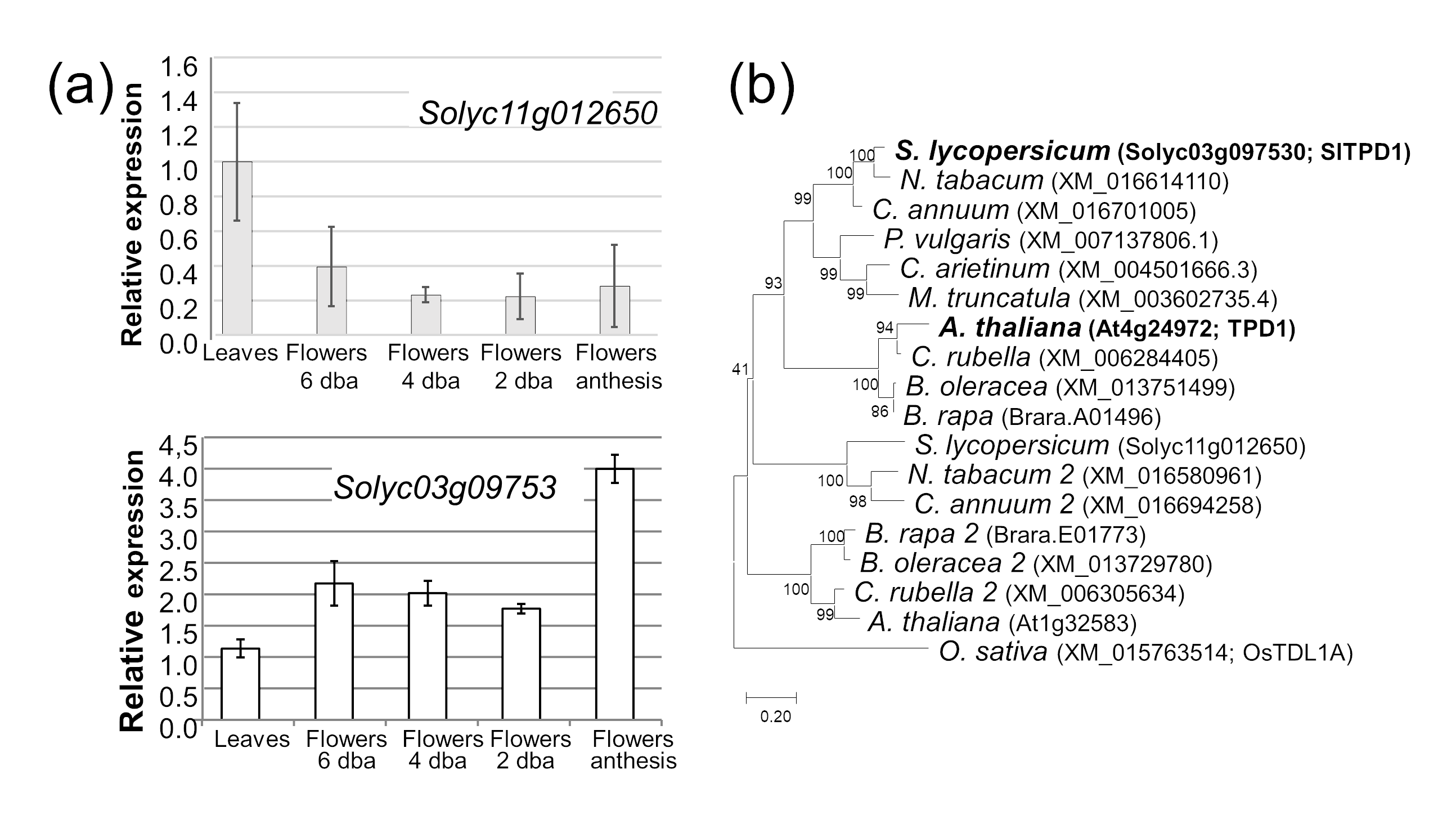

Supplement: Supplementary file 1 — Figure S1. Identification of TPD1‐like genes in tomato. [file TPJ-112-1281-s005.png]

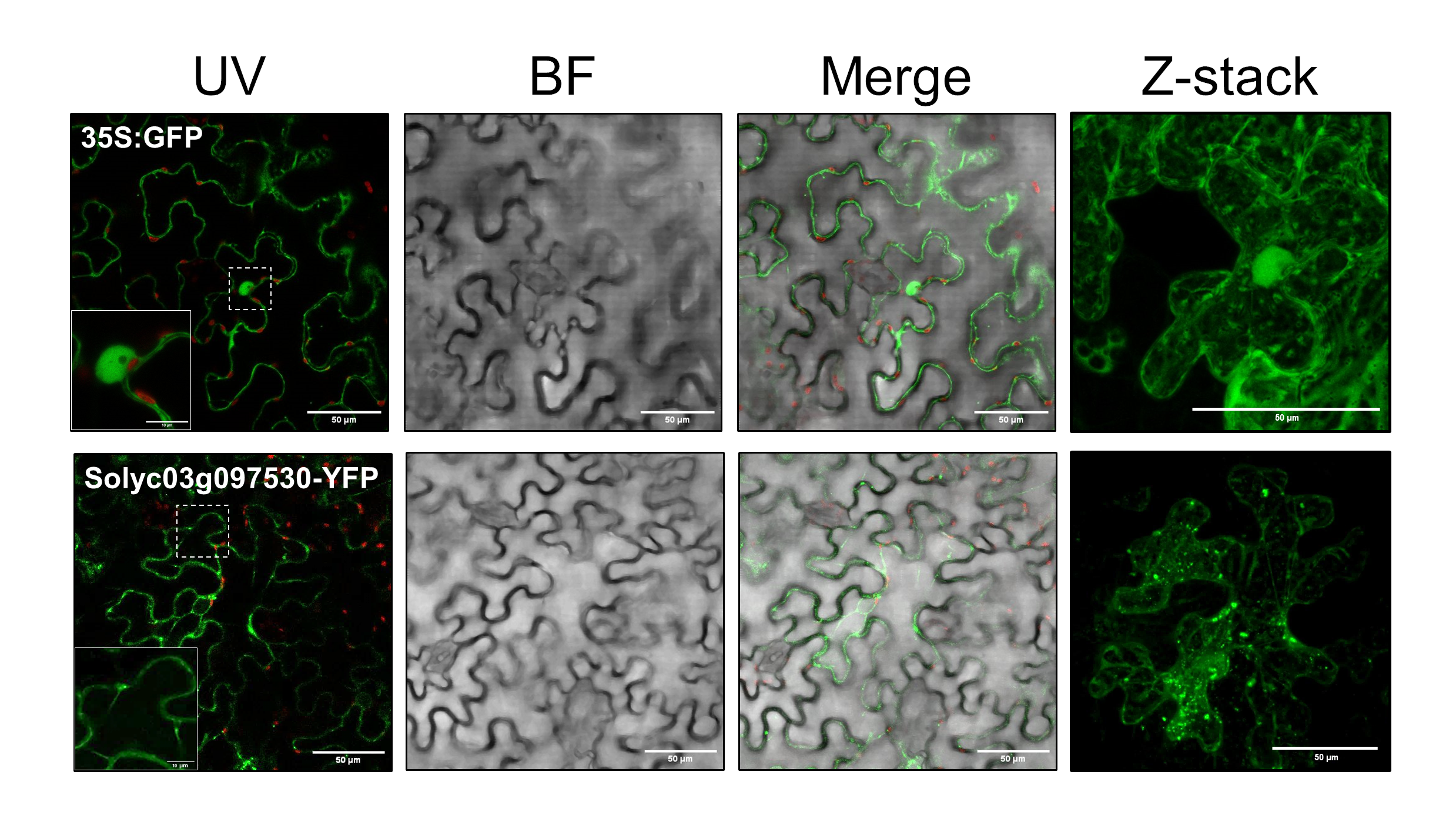

Supplement: Supplementary file 2 — Figure S2. Subcellular localization of Solyc03g097530 (SlTPD1) protein in Nicotiana benthamiana leaves. [file TPJ-112-1281-s006.png]

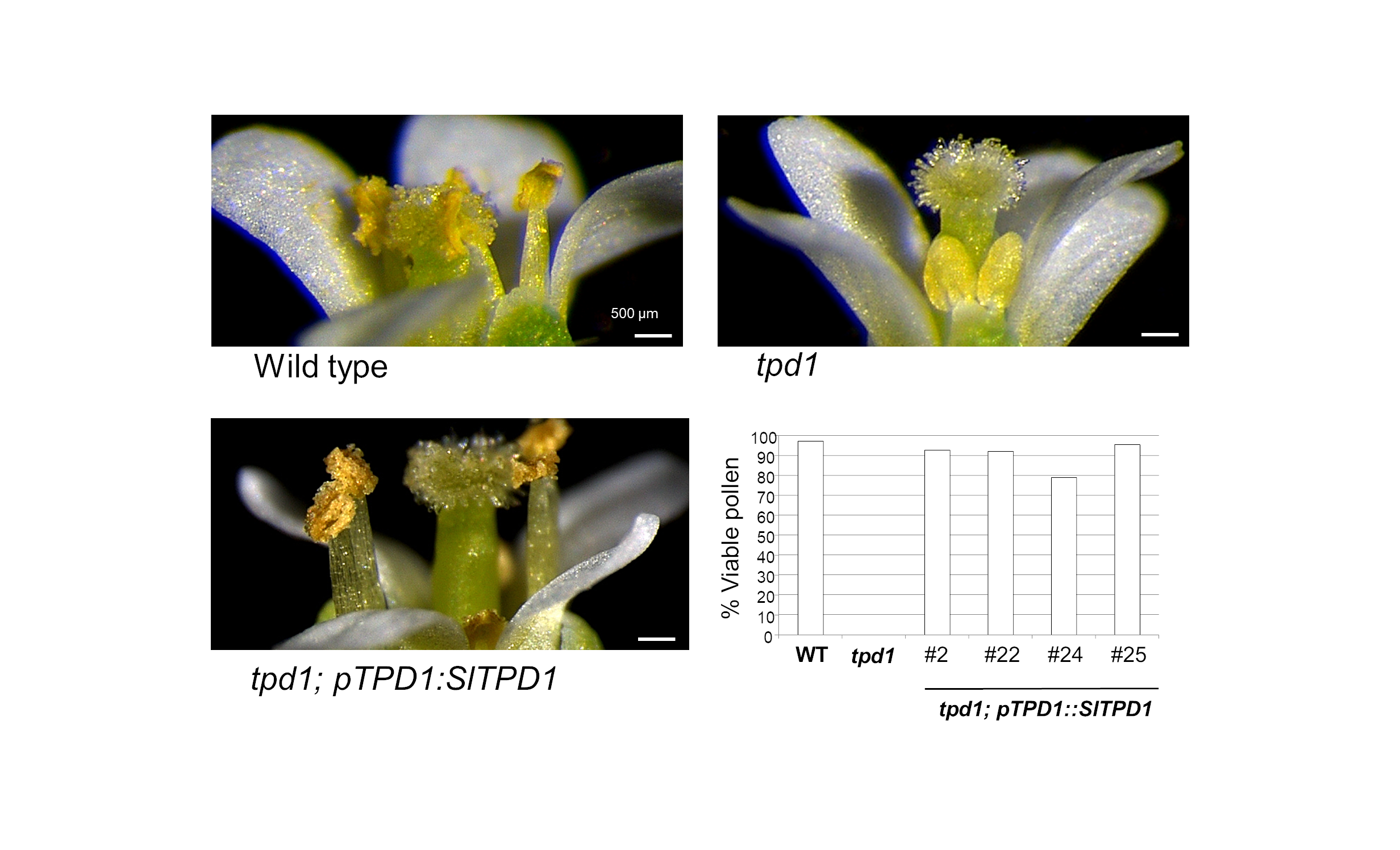

Supplement: Supplementary file 3 — Figure S3. Complementation of the male‐sterile floral phenotype of the Arabidopsis tpd1 mutant using the Solyc03g097530 (SlTPD1) gene. [file TPJ-112-1281-s001.png]

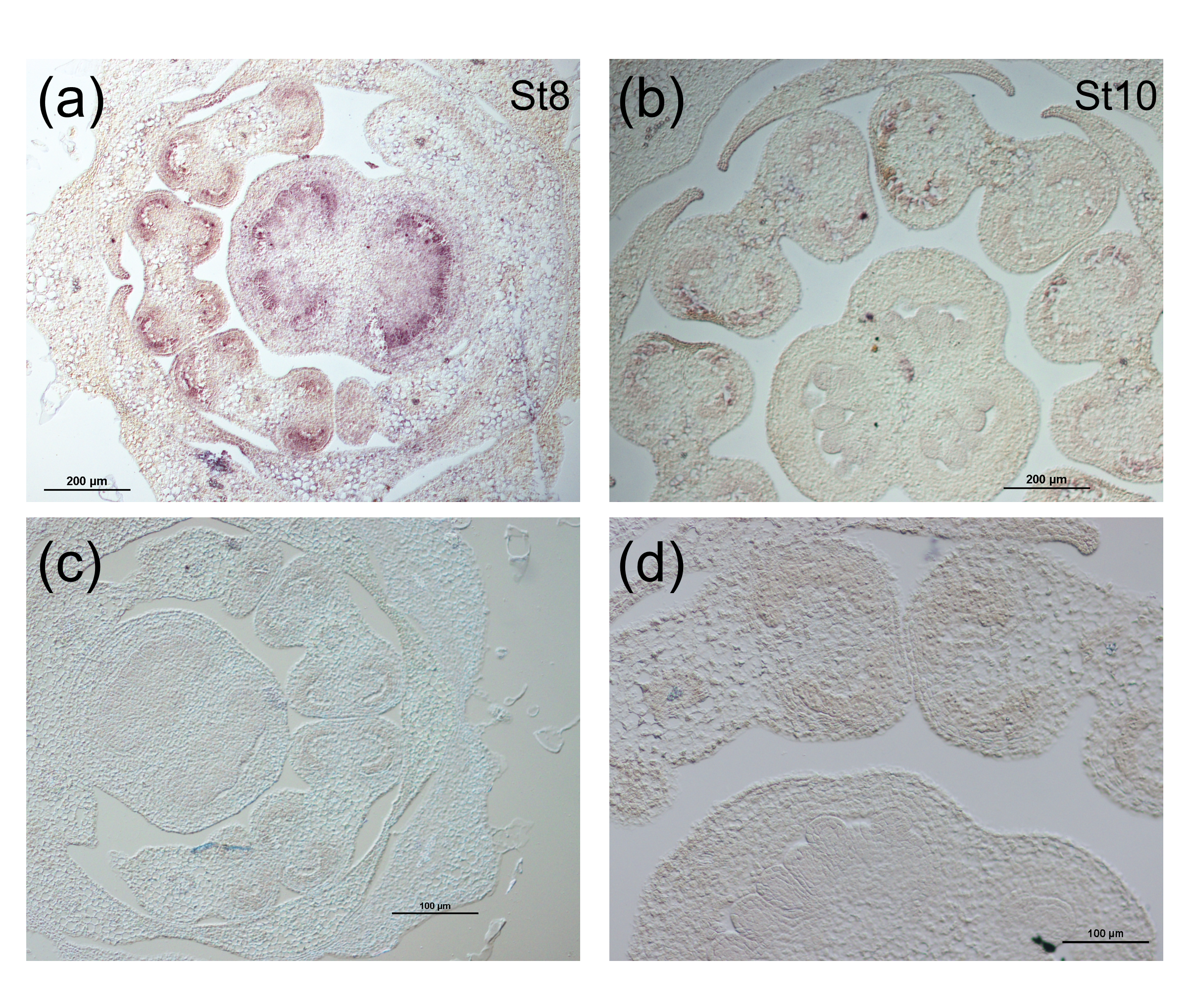

Supplement: Supplementary file 4 — Figure S4. Expression of Solyc03g097530 (SlTPD1) in the ovary detected using in situ hybridization. [file TPJ-112-1281-s003.png]

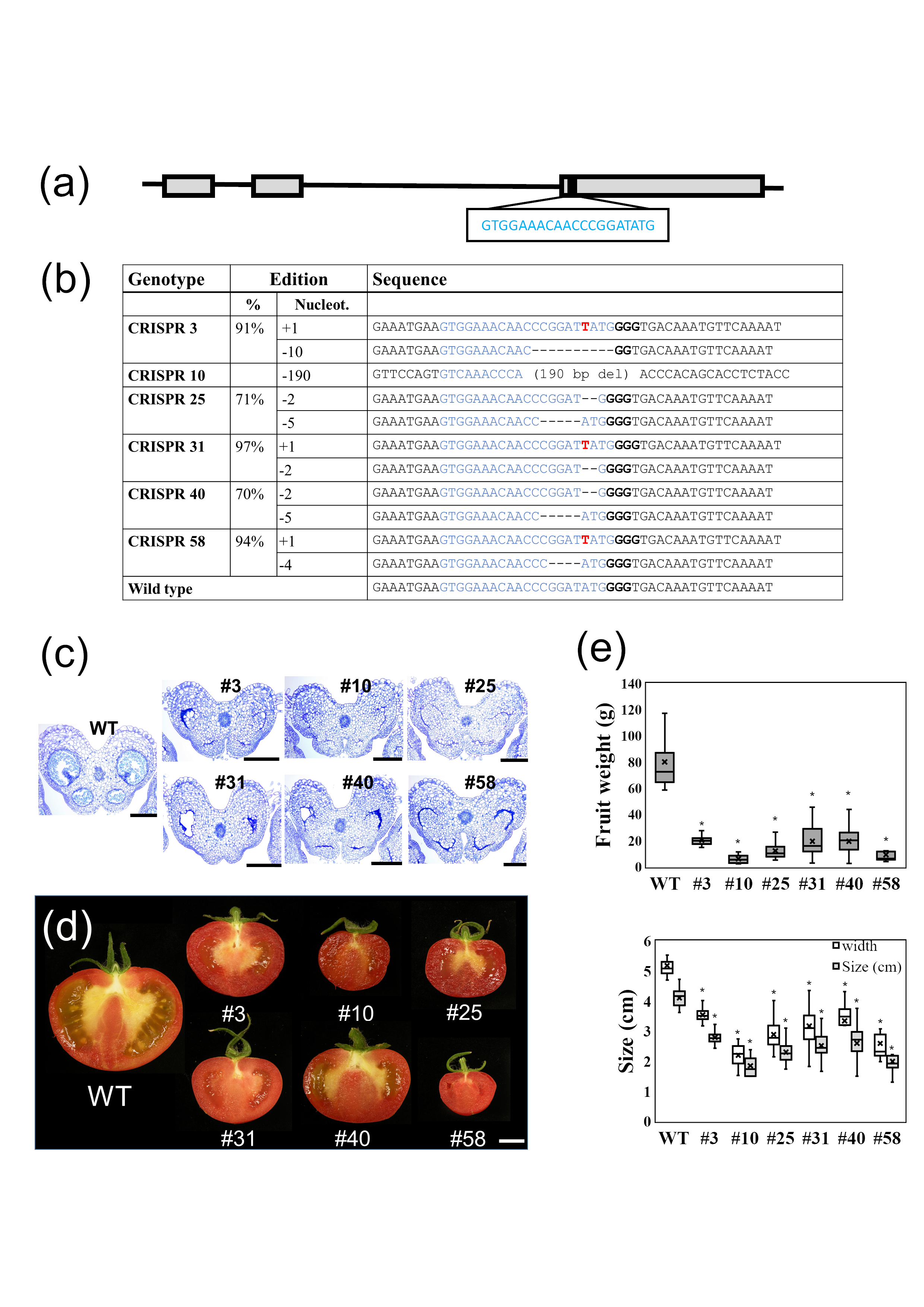

Supplement: Supplementary file 5 — Figure S5. Characterization of CRISP/Cas9‐mediated Solyc03g097530 (SlTPD1) edited tomato plants. [file TPJ-112-1281-s007.png]
