# Supplementary material for: The tapetal tissue is essential for the maintenance of redox homeostasis during microgametogenesis in tomato
Source: Plant J. 2022 Nov 19;112(5):1281–97. doi: 10.1111/tpj.16014 (PMC10100220; doi:10.1111/tpj.16014)
Supplement: Supplementary file 6 — Table S1. Accession numbers of TPD1‐like gene sequences from different plant species used for the phylogenetic analysis. Table S2. Oligonucleotides used in this study. [file TPJ-112-1281-s004.pdf]

**Table S1.** Accession number of *TPD1-like* gene sequences from different plant species used for the phylogenetic analysis.

| Name | Species | Gene ID |
|---|---|---|
| S. lycopersicum Chr3 (SlTPD1) | *Solanum lycopersicum* | *Solyc03g097530* |
| N. tabacum TPD1-like | *Nicotiana tabacum* | XM_016614110 |
| C. annuum TPD1-like | *Capsicum annuum* | XM_016701005 |
| TPD1 | *Arabidopsis thaliana* | *At4g24972* |
| C. rubella TPD1-like | *Capsella rubella* | XM_006284405 |
| B. oleracea TPD1-like | *Brassica oleracea* | XM_013751499 |
| B. rapa TPD1-like | *Brassica rapa* | *Brara.A01496* |
| P. vulgaris TPD1-like | *Phaseolus vulgaris* | XM_007137806.1 |
| C. Arietinum TPD1-like | *Cicer arietinum* | XM_004501666.3 |
| M. truncatula TPD1-like | *Medicago truncatula* | XM_003602735.4 |
| S. lycopersicum Chr11 | *Solanum lycopersicum* | *Solyc11g012650* |
| N, tabacum2 TPD1-like | *Nicotiana tabacum* | XM_016580961 |
| C. annuum2 TPD1-like | *Capsicum annuum* | XM_016694258 |
| C. rubella TPD1-like | *Capsella rubella* | XM_006305634 |
| B. oleracea TPD1-like | *Brassica oleracea* | XM_013729780 |
| B. rapa TPD1-like | *Brassica rapa* | *Brara.E01773* |
| AT1G32583 | *Arabidopsis thaliana* | *At1g32583* |
| OsTDL1A | *Oryza sativa* | XM_015763514 |

**Table S2**. Oligonucleotides used in this study.

| Oligo name | Sequence (5'-3') | GeneID | Experiment |
|---|---|---|---|
| LB1 SAIL | GCCTTTTCAGAAATGGATAAATAGCCTTGCTTCC | | *tpd1-2* mutant genotyping |
| tpd1-LP1 | ATGAACCGACGGCGACTTTT | *At4g24972* | |
| tpd1-RP1 | ACAATTCACCATTTCACGAGG | | |
| SlTPD1G For | TTTCAAGTCTCTCATGTGAAAAGG | *Solyc03g097530* | *Sltpd1* mutant genotyping |
| SlTPD1G Rev | CCATCGTTGACGAGACAATC | | |
| Q-SlTPD1 For | CTCGTATATTCAAGCGCCTACA | *Solyc03g097530* | qRT-PCR experiments |
| Q-SlTPD1 Rev | CAGAGAGTGGGTAACGGAAAG | | |
| Q-SlACT For | GGTATCCACGAGACTACCTACA | *Solyc11g005330* | |
| Q-SlACT Rev | TGCTCATACGGTCAGCAATAC | | |
| Q-SlRbohA For | AACCATACGCTTGGCTCACA | *Solyc01g099620* | |
| Q-SlRbohA Rev | ACGAGCAGCACCAGACTTAC | | |
| Q-SlRbohE For | GCCTCCAAACTCCGCCGGAAAT | *Solyc06g075570* | |
| Q-SlRbohE Rev | CTTCCGATGCTGCTGCCGATGT | | |
| Q-SlRbohG For | GGAGCCATGGACATGCGTCTCG | *Solyc08g081690* | |
| Q-SlRbohG Rev | CCTTGTGTGCGCTGGAACGAGT | | |
| Q-SlGRX For | GTTTGGGCTGGGTGTTGGAC | *Solyc08g036570* | |
| Q-SlGRX Rev | TTTCCACCAACGAACACGGC | | |
| Q-SlTGA9 For | TGGGAGGTGGTACTGCTGCTGG | *Solyc06g074320* | |
| Q-SlTGA9 Rev | TGCAGTTCGAAGCTCGGCTATGT | | |
| Q-SlTGA10 For | GGAGATTTCCGCCCATCCGAGC | *Solyc10g078670* | |
| Q-SlTGA10 Rev | CCAGCCCTTGTGAGAGAGCTTCC | | |
| Q-Off For | GGCCTCGCTCTATTCTATTTAACATG | *Solyc09g089880* | |
| Q-Off Rev | TTTGCAGCTAAACTATAAGCACACCC | | |
| SlTPD1cDNA For | ATGAGCTCTCAGTCATTGAAACG | *Solyc03g097530* | Amplification of *SlTPD1* cDNA |
| SlTPD1cDNA Rev | GCAGACAACAGAAGAAACAGAGAG | | |
| pAtTPD1 For | CCGGGTGGGATACAACATGTTG | *At4g24972* | Amplification of *AtTPD1* promoter (2.660 bp) |
| pAtTPD1 Rev | GTGCGTAGACGTCGAAGAACTAAA | | |
| SlTPD1 CRP For | ATTGTGGAAACAACCCGGATATG | *Solyc03g097530* | CRISPR guide *SlTPD1* |
| SlTPD1 CRP Rev | AAACCATATCCGGGTTGTTTCCA | | |
| SlSDS is for | ATGAAGCGAAAGTTACATGCAGAAG | *Solyc04g008070* | SlSDS probe for in situ hybridization |
| SlSDS is rev | TCAATTACTTTTGCATTTCTATTCCGC | | |
| TomA5B is for | GGTACCACCGAGCGAAGCGTGC | *Solyc01g086830* | TomA5B probe for *in situ* hybridization |
| TomA5B is rev | GACATCAAAATGATAAATATTCA | | |
| SlGRXC9 Fw | ATGCAGGTAGTGAAGGAGTCATC | *Solyc08g036570* | BiFC experiments |
| SlGRXC9 Rv | TTAGAGCCAGAGGGCTCCG | | |
| SlTGA9 Fw | ATGGCGAGTCAAGGAATTGGAG | *Solyc06g074320* | |
| SlTGA9 Rv | TCAGAAATTTGAGAAGTGGTTCTGAG | | |
| SlTGA10 Fw | ATGGGTCTTCAAAGTCATGAAAATCAA | *Solyc10g078670* | |
| SlTGA10 Rv | TTATTCATGTCGAGGCCGAG | | |
